# Quantum-Chemical Design of Molecular Structures of Tetra-, Penta- and Hexanuclear Metal Clusters Containing Aluminum and 3*d*-Element Atoms

**DOI:** 10.3390/ma13081852

**Published:** 2020-04-15

**Authors:** Oleg V. Mikhailov, Denis V. Chachkov

**Affiliations:** 1 Department of Analytical Chemistry, Certification and Quality Management, Kazan National Research Technological University, K. Marx Street 68, 420015 Kazan, Russia; 2 Kazan Department of Joint Supercomputer Center of Russian Academy of Sciences – Branch of Federal Scientific Center “Scientific Research Institute for System Analysis of the RAS”, Lobachevskii Street 2/31, 420111 Kazan, Russia; de2005c@gmail.com

**Keywords:** metal cluster, aluminum, *d*-element, molecular structure, thermodynamic parameters, DFT method

## Abstract

Various data on the structural and thermodynamic characteristics of polynuclear metal clusters containing atoms of aluminum and various *d*-elements with the general formula Al*_n_*M***_m_*** where (*n + m*) is 4, 5, or 6, and which can be precursors for the formation of nanoparticles of elemental metals or intermetallic compounds, have been systematized and discussed. It has been noted that each of these metal clusters in principle is able to exist in very diverse structural isomers, differing significantly among themselves in terms of the total energy and spin multiplicity of the ground state, the number of which is determined by both the specific values of *n* and *m*, and the nature of *d*-elements in their compositions. The presence of very complex dynamics with respect to the changes of the individual thermodynamic characteristics of the metal clusters under consideration as well as the thermodynamic parameters of the reactions of their formation, depending on the nature of the *d*-element, were also ascertained. In the main, the given review is devoted to the authors’ works published over the last 10 years. Bibliography – 96 references.

## 1. Introduction

As is known, micro- and nanoparticles of elemental metals and their compositions, and primarily from among *p*- and *d*-elements, are now very important in modern chemistry and chemical technology. On the one hand, they are a kind of “precursor” for the production of micro- and nanoparticles of metal oxides, metal sulfides, and metal chalcogenides (which, in turn, appear to be very convenient starting materials for producing, for example, ceramic materials, catalytic and sorption systems). On the other hand, they themselves have a number of specific (and very useful) properties from an anthropogenic point of view. There is great interest in this type of nanoparticle, i.e., those that contain two or more different chemical elements in their structural units, because in this case, from purely theoretical considerations, it is very likely that they will have a number of new properties that are not observed in nanoparticles containing atoms of only one chemical element. These nanoparticles are composed of heteronuclear metal clusters with chemical metal–metal bonds formed by identical as well as different atoms. In view of the circumstance just noted, it seems to be an urgent task related to both confirming the very principal possibility of the existence of nanoparticles consisting of various metal elements and having a specific chemical composition, and if confirmed, by revealing all possible structural forms and configurations for them with using modern quantum chemical calculations.

To date, a very significant number of studies have been devoted to heteropolynuclear metal clusters with a diverse number and assortment of metal atoms in structural units—their number is measured in many hundreds, and all of them in this review paper cannot be simply cited. Most of them were devoted to heteropolynuclear metal clusters containing atoms of various 3*d*, 4*d,* and 5*d* elements. In particular, are related the publications [1,2,3,4,5,6,7,8,9,10,11,12,13,14,15,16,17,18,19,20,21,22,23,24,25,26,27,28,29,30,31,32,33,34,35,36,37,38,39,40,41,42,43,44,45,46,47,48,49,50]. Some such metal clusters have been applied in various fields of science and technology (see, f.e., [1,3,20,36,37]). In the works cited above [1,2,3,4,5,6,7,8,9,10,11,12,13,14,15,16,17,18,19,20,21,22,23,24,25,26,27,28,29,30,31,32,33,34,35,36,37,38,39,40,41,42,43,44,45,46,47,48,49,50], the objects of study were the so-called (*dd*)heterobimetallic metal clusters, which included atoms of two different *d*-elements, in particular, (Au, Fe) [9], (Pd, Ag) [12,21,43], (Au, Ag) [26], (Au, Pd) [33], (Cu, Fe) [38] and (Pt, Cu) [39]. However, of no less interest are the (*pd*)heterobimetallic metal clusters that include atoms of different categories of metals, namely, *p*- and *d*-elements, since theoretically it can be expected that they will demonstrate such new properties that are not inherent to metal clusters containing metal atoms of only one category. These metal clusters, however, were considered in a much smaller number of works, in particular, in [51,52,53,54,55,56,57,58,59,60,61,62,63].

Heterometallic metal clusters containing the atoms of those metals that are important in various fields of science and technology, namely aluminum and 3*d* elements (M), are very interesting objects from both a purely academic and practical points of view in this field. Some of them, in particular, Sc_n_Al, Y_n_Al, and Al_n_Ti, were considered in [58,59,60,61,62,63]. Such metal clusters, however, contain only two types of chemical bonds, namely either M–M and Al–M, or Al–M and Al–Al. More interesting for consideration are those (*pd*)metal clusters that contain all three possible chemical bonds here, namely M–M, Al–M, and Al–Al. The simplest of them are tetranuclear metal clusters, where in principle, two different types of geometric bodies are possible, at the vertices of which there are M and Al atoms, namely a quadrangle and a tetrahedron, and structural variations are already quite diverse. However, much greater possibilities in terms of the diversity of molecular structures open up starting from five-atom clusters. At the present time, there is already a number of publications that have examined the structural features of this category of (*pd*)metal clusters consisting of four, five, and six atoms, and the quantum-chemical calculations of these metal clusters were carried out using the density functional method (DFT) combining the standard extended split-valence QZVP basis [64,65] and the OPBE functional [66,67]. To build quantum chemical models of the molecular structures of the metal clusters under examination, GAUSSIAN09 software was used [68]. Moreover, the accordance of the found stationary points to the energy minima was confirmed by calculation of the second derivatives with respect to the atomic coordinates. Further, all equilibrium structures corresponding to the minima at the potential energy surface revealed only real positive frequency values. Parameters of the molecular structures for spin multiplicities (*M_S_*) more than 1, were determined using the so-called unrestricted method (*UOPBE*), for *M_S_* = 1, using so-called restricted method (*ROPBE*). Along with this, the unrestricted method in conjunction with the GUESS = Mix option was used for the cases when *M_S_* was equal to 1. The data obtained as a result of such a procedure, were similar to those obtained using ROPBE method. The data of works [69,70,71,72] give us reason to assert that the given method allows to obtain the most accurate estimation of ratio between energies of the high-spin state and low-spin state and, at the same time, rather reliably predicts the key geometric parameters of molecular structures for various compounds of 3*p*- and 3*d*-elements. That is why the DFT OPBE/QZVP method was used by authors of the given review article in the calculation of molecular structures in all their works [73,74,75,76,77,78,79,80,81,82,83,84,85,86,87,88,89,90,91,92,93,94,95,96], where such (*pd*)metal clusters were considered. The given review paper will be devoted to the systematization and discussion of the main results of those calculations that are presented, namely in these publications.

## 2. Tetranuclear (AlM) Metal Clusters

**Figure 1:**
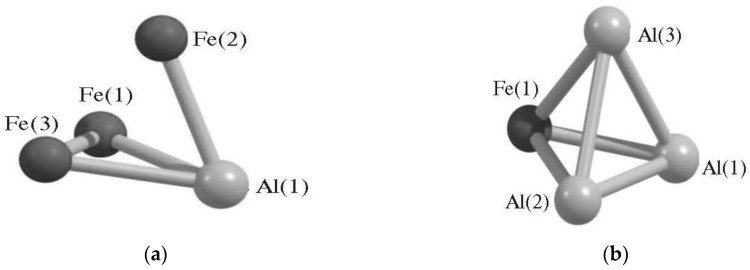
The images of molecular structures of most stable AlFe_3_ (**a**) and Al_3_Fe (**b**) isomers [76].

Tetranuclear metal clusters containing aluminum atoms and *d*-metal atoms M can be divided into three categories depending on the number of both these atoms in the molecule, namely AlM_3_, Al_2_M_2_, and Al_3_M. Currently, information on metal clusters with such stoichiometric compositions is available only for one of the *d*-elements, namely for M = Fe. Such metal clusters were considered by us in the works [73,74,75,76,77,78,79,80]. In the earliest of them [73], a metal cluster of AlFe_3_ composition was described, for which the total number of theoretically possible isomers (4) was revealed in this work, and data on the coordinates of the iron and aluminum atoms included in its composition were presented. Similar information on the total number of isomers of this metal cluster is reported in publications [74,75]. However, a more detailed consideration of this metal cluster and its analogue with the inverse relationship between the numbers of Al and Fe atoms, namely Al_3_Fe, is carried out in [76,77], revealing the presence of 10 isomers of AlFe_3_ and seven isomers of Al_3_Fe. Information on their relative stability is presented in Table 1. In the same works, the parameters of the molecular structures of all these isomers were presented. According to them, in eight isomers of AlFe_3_, aluminum and iron atoms are located at the vertices of a distorted tetrahedron, distorted to one degree or another. In the other two, all four atoms are located in the same plane. It is characteristic that, in any of these ten isomers, all three theoretically possible Al–Fe chemical bonds are present, while three Fe–Fe bonds are present in only two isomers, and in most of them (in seven), only one such bond is realized. In this connection, we should note that, among these ten isomers, there is one in which there are no bonds between the atoms of the above *d*-element. At the same time, curiously, the most stable in energy terms (i.e., having the lowest total energy among all other isomers) is one of those seven isomers in which there is only one Fe–Fe bond [76,77]. According to the data presented in these publications, the ground state of this isomer is the spin quartet. The isomers closest to it in terms of total energy have a doublet and quartet ground state and are almost identical energies, which are only slightly larger than the total energy of the most stable isomer (by 11.2 and 11.3 kJ/mol, respectively). Isomers of the Al_3_Fe metal cluster, despite the closeness of their formal stoichiometric compositions to AlFe_3_ isomers, nevertheless are quite significantly different from AlFe_3_ isomers, not only in their total number, but also in appearance [76,77]. In six of seven isomers of Al_3_Fe, there are all three theoretically admissible Al–Fe chemical bonds, and only one has such a bond. As in the AlFe_3_ metal cluster, there are distorted tetrahedral and planar structures (4 and 3, respectively). However, here, unlike AlFe_3_, the most energy stable isomer contains a complete set of metal – metal bonds (three Al–Fe bonds and three Fe–Fe bonds). Molecular structures of both these isomers are shown in Figure 1. The Al–Fe chemical bond lengths in AlFe_3_ isomers are generally somewhat longer than those in Al_3_Fe, which may be connected with the absence of a complete set of Fe–Fe chemical bonds in most AlFe_3_ isomers [76,77].

**Figure 2:**
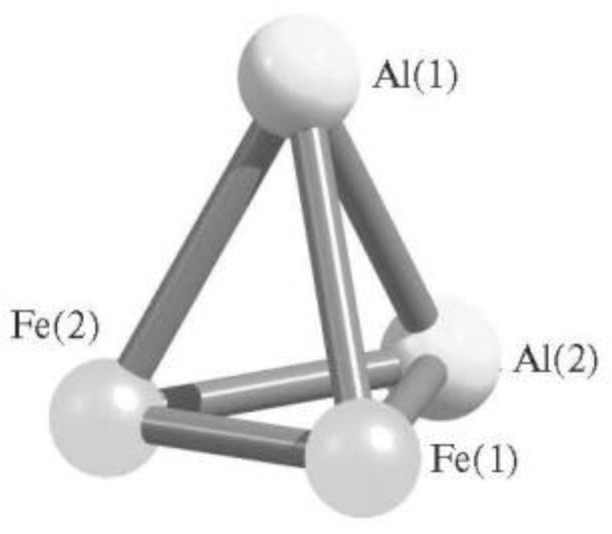
The image of molecular structure of most stable Al_2_Fe_2_ isomer [79].

Another theoretically possible (AlFe) tetranuclear metal cluster, namely Al_2_Fe_2_, was considered in [78,79,80]. Unlike the AlFe_3_ and Al_3_Fe metal clusters, it contains all three of the above types of chemical bonds (M–M, Al–M and Al–Al), and therefore more isomers can be expected here. Indeed, according to the data of [78,79,80], it can exist in 12 different isomers (Table 1), the most energetically favorable of which has the structure of a distorted tetrahedron with the maximum theoretically possible set of chemical bonds Al–Fe and Fe–Fe (three each of these types of bonds) (Figure 2). Outwardly, this isomer resembles the one shown in Figure 1b, but in it all four Al–Fe bonds have the same length (249.5 pm each), while in Al_3_Fe all these bonds are different and generally shorter (234.6, 236.7, and 250.6 pm) [79,80]. For the majority of other isomers of Al_2_Fe_2_ (seven out of 12), as well as for tetranuclear metal clusters already considered in this section (AlFe), the geometry of the distorted tetrahedron is more typical, although flat or almost coplanar structures also occupy a prominent place [78,79,80]. Isomers of Al_2_Fe_2_ with this form, however, have total energy values of more than 100 kJ/mol higher than the total energy of the most low-energy isomer. Further, the total amount of chemical bonds Al–Al, Al–Fe, and Fe–Fe in these isomers ranges from 4 to 6. The first of these bonds is absent only in one isomer, and the third, in two isomers of the metal cluster under examination. It should be noted in this connection that the lengths of the M–M, Al–M, and Al–Al bonds in various isomers of these metal clusters are in such ranges that, given the atomic radii of Al (143 pm) and Fe (126 pm), appear quite natural and predictable. In particular, in Al_3_Fe isomers, Al–Al bond lengths are in the range of 257–277 pm, Al–Fe bond lengths are in the range of 235–255 pm. In AlFe_3_ isomers, Fe–Fe bond lengths are in the range of 207–219 pm, Al–Fe bond lengths are in the range of 238–276 pm. At the same time, most of the valence (bond) angles of these same bonds, as well as the torsion (dihedral) angles, have values substantially less than 90°. The more detailed information on the structural parameters of the most energetically stable of the above metal clusters is presented in Table 2.

The images of molecular structures of all these tetranuclear metal clusters are presented in Supplementary Materials (Figures S1–S3).

**Figure 3:**
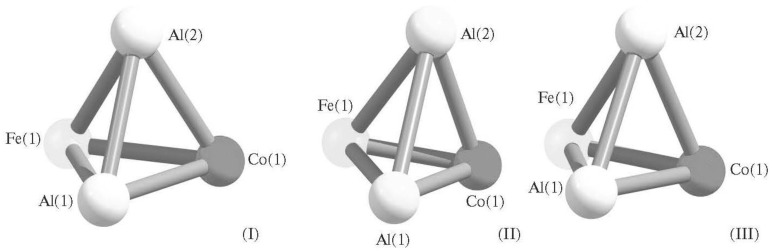
Molecular structures of three Al_2_FeCo isomers: (**I**)—with most high energy, (**III**) —with most low energy and (**II**)—with intermediate energy between (**I**) and (**III**) [79].

In [79,80], a quantum-chemical calculation of the parameters of molecular structures of (heterotri)tetranuclear metal cluster having the composition Al_2_FeCo, was also carried out, for which it was found that only three isomers could be found that look very similar to each other (Figure 3).

These isomers, however, are very different from each other in total energy values as well as the spin multiplicity of the ground state. There is no doubt that (heterobi)tetranuclear metal clusters containing aluminum atoms and atoms of other *d*-elements are also possible, but no information has appeared in the literature on this subject yet. 

## 3. Pentanuclear (AlM) Metal Clusters

**Figure 4:**
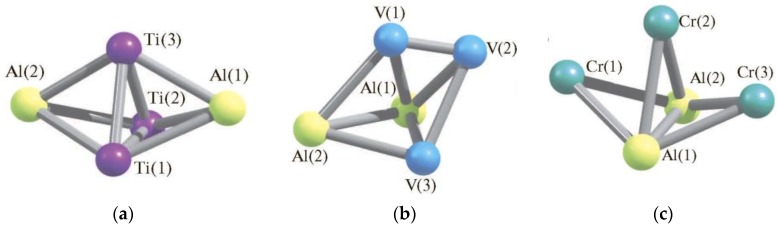

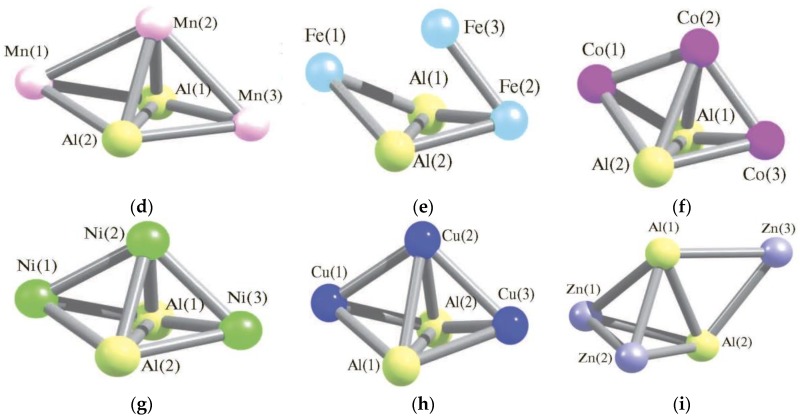
The images of molecular structures of most energetic stable Al_2_M_3_ metal clusters (M—3d-element) [92,93]: (**a**)—Al_2_Ti_3_, (**b**)—Al_2_V_3_, (**c**)—Al_2_Cr_3_, (**d**)—Al_2_Mn_3_, (**e**)—Al_2_Fe_3_, (**f**)—Al_2_Co_3_, (**g**)—Al_2_Ni_3_, (**h**)—Al_2_Cu_3_, (**i**)—Al_2_Zn_3_.

The given type of metal clusters has been analyzed in much more detail than tetrabinuclear ones, and is currently known for all 3*d* elements (except for Sc), as well as for two 4*d* elements (Mo and Ag). It should be noted, however, that all of these metal clusters are of the same type and have the same stoichiometric composition of Al_2_M_3_. Meanwhile, pentabinuclear metal clusters with other theoretically possible sets of aluminum atoms and *d*-element atoms in the molecule (and namely, Al_4_M, Al_3_M_2_ and AlM_4_) have not yet been considered. The specifics of molecular structures and thermodynamic characteristics of such metal clusters having the composition indicated above, where M is a 3*d* element, are discussed in [74,75,81,82,83,84,85,86,87,88,89,90,91]. We should note immediately that, according to the calculation data by the DFT method, for each of these M, there is an individual set of isomers, both in assortment and in their total number (*N*), which varies over a very wide range—from 7 (in the case of Al_2_Ni_3_) to 25 (in the case of Al_2_Mn_3_) (Table 3). The relative total energies of these isomers also vary over a very wide range (Table 4). The most energetically advantageous among these isomers for the above M are shown in Figure 4. The molecular structure parameters of these isomers are given in Table 5. A complete assortment of molecular structures of all metal clusters considered by us can be found in the publications [74,75,81,82,83,84,85,86,87,88,89,90,91] cited above, and in the Supplemental Materials.

According to data presented in [81,83], of the 14 identified isomers of the Al_2_Ti_3_ metal cluster, only Al–Al valence bonds are realized in only seven of them, namely, Al_2_Ti_3_ (I), Al_2_Ti_3_ (II), Al_2_Ti_3_ (IV), Al_2_Ti_3_ (V), Al_2_Ti_3_ (VII), Al_2_Ti_3_ (VIII), and Al_2_Ti_3_ (X), the direct valence bonds Al–Ti and Ti–Ti, at least in the singular, each occur in all these metal clusters. The most favorable in terms of energy is the Al_2_Ti_3_ (XI) isomer with spin multiplicity *M_S_* = 5 and the geometry of the trigonal bipyramid (Figure 4a), in the “equatorial plane” of which there are three titanium atoms, the aluminum atoms are located at its vertices. The Al_2_Ti_3_ (XII) isomer with *M_S_* = 3 following the increase in total energy, has a similar structure. Four of these isomers, namely Al_2_Ti_3_ (IV), Al_2_Ti_3_ (V), Al_2_Ti_3_ (IX), and Al_2_Ti_3_ (XI), have a spin multiplicity 5, the same, namely, Al_2_Ti_3_ (I), Al_2_Ti_3_ (VI), Al_2_Ti_3_ (X), Al_2_Ti_3_ (XII), spin multiplicity 3. In the remaining six, the spin singlet turns out to be the ground state. As it may be seen, the spin state with *M_S_* = 1 for the metal cluster under consideration is predominant, although in fairness, it should be noted that the closest in energy to the Al_2_Ti_3_ (XI) isomer are the Al_2_Ti_3_ (XII) and Al_2_Ti_3_ (V) isomers with relative energies of 12.6 and 19.7 kJ/mol, respectively, exceeding the energy of Al_2_Ti_3_ (XI), having *M_S_* = 3 and 5, respectively (Table 4).

In [82,83], a quantum chemical calculation of the Al_2_V_3_ metal cluster was carried out. Information on the relative energy of its isomers is presented in Table 4. As can be seen from Table 4, in six out of 11 isomers of this metal cluster, namely in Al_2_V_3_ (I)–Al_2_V_3_ (VI), there is a direct Al–Al valence bond, while in the other five, it is absent and only V–V and V–Al bonds take place in them. Moreover, that is noteworthy, in two structures, namely Al_2_V_3_ (VIII) and Al_2_V_3_ (IX), only chemical bonds between atoms of different chemical elements (i.e., V–Al) take place, while between the same atoms there are none (which, by the way, in none of the Al_2_Ti_3_ metal clusters described above is observed). As in the case of the Al_2_Ti_3_ metal cluster, its most stable isomer, namely Al_2_V_3_ (V), has not the highest spin multiplicity (6), but a lower (though not the lowest) (4). At the same time, that is characteristic, structures with the highest spin multiplicity 6 as a whole have noticeably higher values of the total energies than structures with multiplicities 2 and 4. In addition, the Al_2_V_3_ (II) structure closest in energy to Al_2_V_3_ (V) possesses the same spin multiplicity, and its molecular structure resembles the structure of Al_2_V_3_ (V), at least in general terms. At the same time, the following three structures with increasing energy, namely, Al_2_V_3_ (III), Al_2_V_3_ (I), and Al_2_V_3_ (IV), have different values of spin multiplicity, namely, 6, 2, and 2, respectively. The most unstable is the isomer Al_2_V_3_ (IX), the total energy of which is not only much higher (almost 150 kJ/mol) compared with that for Al_2_V_3_ (V), but also all other isomers of the metal cluster under consideration, which is noteworthy, as it has the highest spin multiplicity (6) [82,83].

For the Al_2_Cr_3_ metal cluster, 20 different isomers were found (Table 3). In thirteen of them, namely, in Al_2_Cr_3_ (I), Al_2_Cr_3_ (III)–Al_2_Cr_3_ (XII), Al_2_Cr_3_ (XVI) and Al_2_Cr_3_ (XVII), there is a direct valence Al–Al bond, while in the other seven it is absent in only Cr–Cr and Cr–Al bonds are realized in them [84,85]. On the other hand, Cr–Cr bonds are present in only six isomers: Al_2_Cr_3_ (I), Al_2_Cr_3_ (V), Al_2_Cr_3_ (VI), Al_2_Cr_3_ (X), Al_2_Cr_3_ (XIII) and Al_2_Cr_3_ (XIX). Further, with the last three isomers, chromium atoms are paired together. In three structures, namely Al_2_Cr_3_ (II), Al_2_Cr_3_ (XIV), and Al_2_Cr_3_ (XV), only chemical bonds between atoms of different chemical elements (i.e., Cr–Al) take place, but there are no bonds between identical atoms (i.e., Al–Al and Cr–Cr). Information on the relative energy of these isomers is given in Table 4. As can be seen from Table 4, the most stable isomer of this metal cluster, Al_2_Cr_3_ (III), also does not have the highest spin multiplicity (7), but is somewhat lower (5). Incidentally, structures with a higher spin multiplicity 7 in general also have larger total energies than structures with a multiplicity of 5 (although smaller than structures with a multiplicity of 1 and 3) [84,85]. It is interesting that this isomer is the only one among the most stable isomers of Al_2_M_3_ in which there are no M–M bonds (Figure 4c), because in all the most stable isomers of the given composition of metal clusters formed by M atoms of the other 3*d* elements, at least one such a bond occurs (see Figure 4a,b,d–i).

The Al_2_Mn_3_ metal cluster is able to exist in 25 different isomers [86]. This amount is the largest among all Al_2_M_3_ metal clusters formed by 3*d* element atoms (Table 4). Further, only 10 of the structures, namely Al_2_Mn_3_ (I)–Al_2_Mn_3_ (VII), Al_2_Mn_3_ (IX), Al_2_Mn_3_ (XII) and Al_2_Mn_3_ (XIV) contain a covalent Al–Al bond, whereas the other 15 ones, Al_2_Mn_3_ structures contain only the Mn–Mn and Mn–Al bonds, and the Al–Al one is absent. In most of these isomers (16 of 25) there are 6 Mn-Al bonds, in six [Al_2_Mn_3_ (I)–Al_2_Mn_3_ (V), Al_2_Mn_3_ (XIV)]–5, and in three [Al_2_Mn_3_ (IX), Al_2_Mn_3_ (X) and Al_2_Mn_3_ (XIII)] by 4. Finally, in most isomers (15 of 25) there is a complete set of Mn-Mn links (by 3). In seven isomers [Al_2_Mn_3_ (VI)–Al_2_Mn_3_ (VIII), Al_2_Mn_3_ (XI)–Al_2_Mn_3_ (XIII), Al_2_Mn_3_ (XXV)], there are two such bonds and in three isomers [Al_2_Mn_3_ (I), Al_2_Mn_3_ (II) and Al_2_Mn_3_ (XXIV)]–by one [86]. The most energetically favorable Al_2_Mn_3_ (VI) isomer has the spin multiplicity of ground state *M_S_* = 6, and contains maximal number of Al–Al and Al–Mn bonds [although number of Mn–Mn in it is lesser than maximal possible number of such bonds (3)] (Figure 4d). The isomer Al_2_Mn_3_ (XX) has relative total energy only by 1.4 kJ/mol higher than total energy of Al_2_Mn_3_ (VI) isomer and the same multiplicity (6). Its geometric configuration is similar to Al_2_Mn_3_ (VI), however, unlike Al_2_Mn_3_ (VI), Al–Al bonds are absent in it [86]. The next isomer with the largest relative total energy, Al_2_Mn_3_ (XXI) (2.2 kJ/mole), is outwardly similar to Al_2_Mn_3_ (XX) but the bond lengths Al–Mn and Mn–Mn as well as a distance between Al1 and Al2 in it are bit longer than in Al_2_Mn_3_ (XX), and *M_S_* of its ground state is 4. The most low-energetic isomer of the given metal cluster having *M_S_* = 2, and namely Al_2_Mn_3_ (XIII), has relative total energy 29.3 kJ/mole and, that characteristically, has the smallest number of metal–metal bonds among all Al_2_Mn_3_ clusters under examination (only 6). Among them, 10 isomers have *M_S_* = 6, eight have *M_S_* = 4, and seven ones have *M_S_* = 2 (Table 4). As can be seen, the low-spin state is not characteristic for such metal clusters, which is quite expected from the ground state of manganese atom (3*d*^5^4*s*^2^ with five unpaired electrons). It is interesting that the most high-energetic isomer of the given metal cluster, and namely Al_2_Mn_3_ (XVIII), has relative total energy, equal to 149.2 kJ/mole, has the structure of trigonal pyramid as the energetically favorable isomer Al_2_Mn_3_ (VI), and exactly the same spin multiplicity of the ground state (Table 4). Most isomers of Al_2_Mn_3_, including the most energetically favorable Al_2_Mn_3_ (VI) which is shown in Figure 4d, have molecular structures resembling a trigonal pyramid. Exceptions to this include only Al_2_Mn_3_ (I), Al_2_Mn_3_ (II), Al_2_Mn_3_ (IX), Al_2_Mn_3_ (X), Al_2_Mn_3_ (XIII), and Al_2_Mn_3_ (XXIV) [86].

The next Mn 3*d* elements of groups VIII, IX, and X of the periodic system of chemical elements, namely Fe, Co, and Ni, however, form a much smaller number of isomers of Al_2_M_3_ metal clusters than Mn. In the case of the first of them, iron, judging by the data presented in [87,88,89], only 8 isomers are realized, in six of which, namely Al_2_Fe_3_ (I) - Al_2_Fe_3_ (VI), there is a direct Al–Al valence bond, while in the other two structures [Al_2_Fe_3_ (VII) and Al_2_Fe_3_ (VIII)], such a bond is absent and they contain only Fe–Fe and Fe–Al bonds. The relative energies of the isomers of this metal cluster are presented in Table 4. It is noteworthy that, for clusters of this stoichiometric composition, the most energy-stable Al_2_Fe_3_ (II) structure (something like a “one-cap” tetrahedron) has multiplicity of 3 which is an intermediate between multiplicities of the high-spin and low-spin states. In the given isomer, only one M–M bond is present (Figure 4e). The Al_2_Fe_3_ (III) structure closest in energy to it with a similar geometric configuration (total energy of which is 12.8 kJ/mol higher than the total energy of the Al_2_Fe_3_ (II) structure) has spin multiplicity 5. The other two structures with *M_S_* = 3, namely Al_2_Fe_3_ (V) and Al_2_Fe_3_ (VII), have relative total energies of 24.1 and 27.1 kJ/mol higher than the structure of Al_2_Fe_3_ (II), while the two structures with *M_S_* = 5, namely, Al_2_Fe_3_ (VI) and Al_2_Fe_3_ (VIII), in terms of relative total energies (31.1 and 24.7 kJ/mol) are only slightly less stable in energy terms. The low-spin state is uncharacteristic for these clusters (Table 4), which is understandable if we take into account the presence of the iron atom in the ground state of the 3*d*^6^4*s*^2^ electronic configuration with four unpaired electrons.

The Al_2_Co_3_ metal clusters are the subject of works [87,88]. The total number of isomers of this metal cluster in comparison with Al_2_Fe_3_ turns out to be somewhat larger (9). However, their structural diversity is noticeably less than that of Al_2_Fe_3_ metal clusters [87,88]. Here, the direct valence bond Al–Al is also realized in six isomers, namely, Al_2_Co_3_ (I)–Al_2_Co_3_ (III) and Al_2_Co_3_ (VII)–Al_2_Co_3_ (IX), while in the other three isomers, namely, Al_2_Co_3_ (IV)–Al_2_Co_3_ (VI), such a relationship is absent. The most stable in terms of energy among all these isomers is Al_2_Co_3_ (III) (Table 4), which represents a trigonal bipyramid, both of whose vertices are occupied by Co atoms (Figure 4f). The spin multiplicity of its ground state is 6. The closest to it in energy (28.9 kJ/mol higher) isomer of Al_2_Co_3_ (IX) also has a trigonal bipyramidal structure, but, in contrast to Al_2_Co_3_ (III), there are at the vertices of this bipyramid Al and Co atoms. It has the same spin multiplicity as Al_2_Co_3_ (III). All other isomers of the Al_2_Co_3_ metal cluster, in principle, are capable of self-existence, and have significantly higher total energies in comparison with Al_2_Co_3_ (III) and Al_2_Co_3_ (IX). The least stable among them is the trigonal bipyramidal Al_2_Co_3_ (V) with two Al atoms at the vertices of the bipyramid, and not connected by a chemical bond. As in the case of the Al_2_Fe_3_ metal cluster, the low-spin state also turns out to be uncharacteristic.

The Al_2_Ni_3_ metal cluster is represented by the smallest number of structural isomers among all other metal clusters—only seven [87,88]. The direct Al–Al valence bond in aluminum–nickel Al_2_Ni_3_ clusters, as well as in aluminum–iron and aluminum–cobalt analogous stoichiometric compositions, is realized again in six of its isomers. The only exception to this is the Al_2_Ni_3_ (VII) isomer. The relative energies of these structures are shown in Table 4. It is noteworthy that the aforementioned isomer of Al_2_Ni_3_ (VII) is also the least advantageous in terms of energy, and the absolute value of its relative energy is much larger than the same indicator for the other six isomers of Al_2_Ni_3_. As for the most advantageous structure in terms of energy, Al_2_Ni_3_ (II) outwardly resembles the Al_2_Co_3_ (III) structure, the same trigonal bipyramid with two M atoms at the vertices (Figure 4g), but with spin multiplicity of the ground state equal to 3 (as in the case of the most energetically favorable structure of the iron–aluminum cluster Al_2_Fe_3_ (II)). The isomer nearest to it in energy, namely Al_2_Ni_3_ (I) with a similar geometric configuration, has a total energy that is 48.6 kJ/mol higher than the energy of the structure Al_2_Ni_3_ (II). Despite the fact that the ground state of the nickel atom (3*d*^8^4*s*^2^) is characterized by the presence of only two unpaired electrons, the low-spin state for Al_2_Ni_3_ clusters, judging by the data of [87,88], is also uncharacteristic as for aluminum–cobalt and aluminum–iron clusters of a similar stoichiometric composition. In fairness, it is worth noting that a high-spin ground state is characteristic for the Al_2_Ni_3_ metal clusters to an even lesser extent, because, as it is easily seen from the data in Table 4, the relative energies of the Al_2_Ni_3_ isomers having *M_S_* = 5, as a rule, significantly exceeds the relative energies of the isomers having *M_S_* = 3.

The next sequence number after the triad (Fe, Co, Ni) 3*d* element, namely Cu, forms the same number of isomers of the Al_2_M_3_ metal cluster as Fe (i.e., 8). In seven of eight of these isomers, the direct valence bond Al–Al is realized. The only exception here is the isomer of Al_2_Cu_3_ (II) [89,90,91]. Also, in 7 out of 8 isomers, with the exception of only Al_2_Cu_3_ (V), there is at least one Cu–Cu bond, and Al–Cu bonds occur in each of these isomers. The following circumstance attracts attention in that the aluminum–copper Al_2_Cu_3_ metal clusters have a very significant total number of metal–metal bonds: from seven to nine. In this connection, it is worth noting that in three of the eight Al_2_Cu_3_ isomers, the total number of metal–metal bonds is only one less than the maximum possible number of Al_2_Cu_3_ compounds, namely 10. The relative energies of all Al_2_Cu_3_ isomers are presented in Table 4. From the data presented in Table 4, it can be seen that the most stable isomer is Al_2_Cu_3_ (I); the total number of metal–metal bonds in this isomer is 9 (Figure 4h). As in the majority of metal clusters having Al_2_M_3_ stoichiometric composition and already considered above, this isomer has not the highest spin multiplicity (4), but lower (2). However, isomers with *M_S_* = 4, namely Al_2_Cu_3_ (II), Al_2_Cu_3_ (IV) and Al_2_Cu_3_ (VI) as a whole, have significantly higher total energies than isomers with *M_S_* = 2 (Table 4).

In [86], a quantum chemical calculation of the Al_2_Zn_3_ metal daster was performed and it was shown that it can exist in 14 different isomers. Relative total energies of these isomers are presented in Table 3. As may be seen from these data, in 10 out of 14 of these isomers, the direct valence bond of Al–Al occurs. The exceptions are the Al_2_Zn_3_ (X)–Al_2_Zn_3_ (XII) and Al_2_Zn_3_ (XIV) isomers. Also, in the each of them, there are at least one Zn–Zn and four Al–Zn bonds. The isomers of Al_2_Zn_3_ metal clusters under examination have a very significant total number of metal-metal bonds, from seven to nine such bonds. In addition, in eight out of 14 isomers, the total number is only one less than the maximum possible number of them in Al_2_M_3_ (10) compounds. In this indicator, Al_2_Zn_3_ is superior to any other of the number of Al_2_M_3_ metal clusters formed by 3*d* element atoms. However, in the most stable isomer, namely Al_2_Zn_3_ (I), the total number of metal–metal bonds is eight (Figure 4i), i.e., nine less than occurs in most isomers of the given metal cluster. Despite of the fact that, for this metal cluster, in principle, isomers with spin multiplicities of the ground state 1, 3, 5, and 7 are possible, in metal clusters discovered as a result of our quantum-chemical calculation, only two values of spin multiplicity for the ground state, namely 1 and 3, take place for Al_2_Zn_3_ isomers. The most stable of them, namely Al_2_Zn_3_ (I), has *M_S_* = 3. Interestingly, the same *M_S_* values have the next two lower-energetic isomers, namely Al_2_Zn_3_ (XIV) and Al_2_Zn_3_ (IV) having relative total energies are 3.2 and 11.0 kJ/mole, respectively. Nevertheless, the most of isomers of the cluster under examination (9 of 14) have spin singlet as ground state (Table 4). The total energy of the isomers *M_S_* = 1 as a whole is much larger than the total energy of the isomers with *M_S_* = 3. The lowest-energy of them Al_2_Zn_3_ (III) differs in energy from the isomer of Al_2_Zn_3_ (I) by 14.0, the most high-energy, Al_2_Zn_3_ (VIII), by 79.2 kJ/mole [86].

The images of molecular structures of the most stable of each of Al_2_M_3_ metal clusters under study and geometric parameters of these structures are also presented in articles [92,93] (see Figure 4 and Table 5, respectively). With respect to the most general structural features of these five-atomic metal clusters containing two Al atoms and three atoms of the 3*d* element M in the structural unit, it should be said that, in full accordance with theoretical expectations, for most of them, the trigonal bipyramid is most typical structure. Moreover, other geometric bodies are not uncommon here, in particular, a tetragonal pyramid and even flat polygons. Most of them are also characterized, on the one hand, by the presence of several metal–metal bonds formed by the same atom with their “neighbors,” and on the other hand, by relatively high values of the lengths of these bonds, which usually exceed 200 pm. Exceptions occur only in a few cases, in particular, in the Al_2_V_3_ metal cluster for bond lengths V1V2 in the isomers Al_2_V_3_ (I)–Al_2_V_3_ (VI), Al_2_V_3_ (X) and Al_2_V_3_ (XI), lying in the range from 171.7 pm [in the Al_2_V_3_ (VI) structure] up to 188.1 pm [in the structure of Al_2_V_3_ (I)]. On the other hand, in general, as expected, the Al–Al bonds are the longest, the M–M bonds are the shortest, while the M–Al bond lengths occupy an intermediate position between the bond lengths formed by two aluminum atoms and two atoms M. Further, the lengths of the M–M, Al–M, and Al–Al bonds in various isomers of these metal clusters are in the ranges that, taking into account the atomic radii of Al (143 pm) and M (132 pm (Ti), 134 pm (V), (Cr), 127 pm (Mn), 126 pm (Fe), 125 pm (Co), (Ni), 128 pm (Cu) and 138 pm (Zn)) seem to be quite natural and predictable. So, in Al_2_Ti_3_ isomers, the lengths of Ti–Ti bonds are in the range 210–260 pm, the lengths of Al–Ti and Al–Al bonds are in the ranges 252–270 pm and 255–280 pm, and in the Al_2_V_3_ isomers are in the ranges 170–275 pm, 250–270 pm, and 255–270 pm respectively. Most of the bond angles between the lines of these bonds and the torsion (dihedral) angles in the isomers of all Al_2_M_3_ metal clusters under consideration have values substantially less than 90°. The vast majority of such metal clusters either do not have symmetry elements at all, or have only one plane of symmetry. One of the few exceptions is the Al_2_V_3_ (IX) metal cluster isomer which has one third-order symmetry axis, three second-order symmetry axes, three symmetry planes, and a centre of symmetry [82]. It is noteworthy that, among the most stable metal clusters of each of the 3*d*-elements considered by us and presented in Figure 4, there is not one having a similar set of symmetry elements.

The images of molecular structures of all these pentanuclear metal clusters are presented in Supplementary Materials (Figures S4–S12).

In the articles [92,93], the thermodynamics of Al_2_M_3_ metal clusters formed by atoms of 3*d* elements was considered and calculation of standard thermodynamic formation parameters [Δ_f_*H*^0^(298 K), Δ_f_*S*^0^(298 K) and (Δ_f_*G*^0^(298 K)], as well as standard enthalpy, entropy and Gibbs energy of reactions of their formation from mono-atomic particles in the gas phase (Δ_f_*H*^0^_298_, Δ_f_*S*^0^_298_, Δ_f_*G*^0^_298_) was carried out. These parameters are presented in Table 6. As can be seen from these data, all Δ_f_*H*^0^(298 K), Δ_f_*S*^0^(298 K) and (Δ_f_*G*^0^(298 K) values are positive, indicating the impossibility of the formation of these metal clusters from simple substances formed by the constituent elements and existing under standard conditions (i.e., from Al (crystal) and corresponding M (crystal)). However, for the reactions of formation of Al_2_M_3_ in gas phase according to general Equation (1),

2Al (gas) + 3M (gas)→Al_2_M_3_ (gas) (1)

another situation takes place. Each of the reactions in Equation (1) is exothermic and belong to processes whose course is due to the enthalpy factor since for any of them, the standard enthalpy is Δ*H*_298_ < 0, and Δ*S*_298_ < 0 (Table 6). In addition, heteronuclear metal clusters formed as a result of such reactions, are characterized by rather high thermal stability. In the publications [92,93], the availability of a very complex dynamics of changes in both the individual thermodynamic characteristics of the Al_2_M_3_ metal clusters under consideration and the thermodynamic parameters of these reactions of their formation depending on the nature of the 3*d* element was also noted. Since for each of them, Δ*S*_298_ is also negative, in the simplest version, according to the Gibbs–Helmholtz equation for the isobaric process (2)

**Figure 5:**
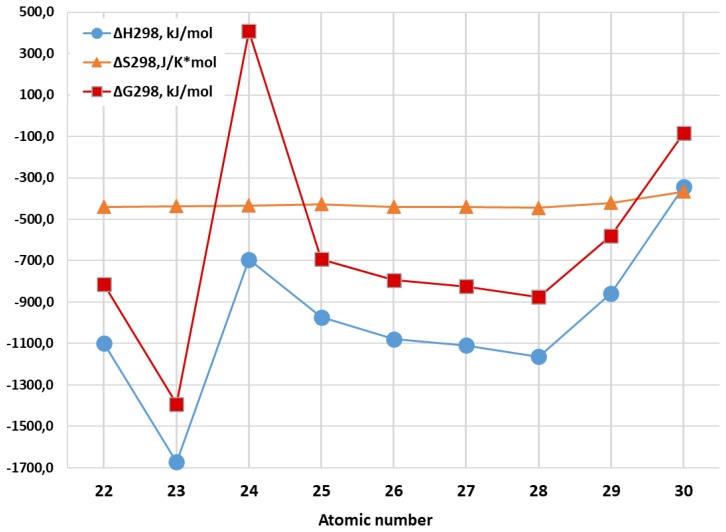
The dependences of the thermodynamic parameters of the reaction (1) of 3*d*-element atomic number.

Δ*G*(*T*) = Δ*H*_298_ – *T*Δ*S*_298_ (2)

where Δ*H*_298_ and Δ*S*_298_ are the enthalpy and entropy changes as a result of the chemical process, referred to standard conditions, *T* is the process temperature in K, and Δ*G*(*T*) is the dependence of the Gibbs free energy on temperature *T* for reaction (1). Regardless of the nature of the 3*d* element M, the values of both of these parameters are negative, and this fact in turn means that the given reaction is thermodynamically resolved at relatively low temperatures and forbidden at high ones. On the contrary, the reverse reaction (1) will be allowed at sufficiently high temperatures and forbidden at low ones. The minimum temperature at which this reverse reaction begins, can be considered as the temperature of the beginning thermal decomposition of the metal cluster (*T*_td_) in the gas phase to individual Al and M atoms. As may be seen from the data presented in the Table 6, the Δ*H*_298_ value and, correspondingly, thermal effect of reaction (1) is very significant in all cases. Further, as it is easy to show with using Equation (2) [92,93], the temperature at which reverse reaction (1) will be possible, for almost all Al_2_M_3_ metal clusters (for with the exception of Al_2_Zn_3_ only) exceeds 1000 K (Table 7). Herewith, Al_2_V_3_ is the most stable in this respect among all the compounds under consideration, Al_2_Zn_3_ is the least stable. Upon transition from Ti to V, the temperature of the beginning of thermal decomposition increases, from V to Cr it decreases, from Cr to Ni it increases and from Ni to Zn it decreases again (Table 7). However, the dynamics of changes in the standard thermodynamic formation parameters (Δ_f_*H*^0^(298 K), Δ_f_*S*^0^(298 K) and Δ_f_*G*^0^(298 K)) of the Al_2_M_3_ metal clusters under consideration is of a somewhat different and more complex character. So, for Δ_f_*H*^0^(298 K) и (Δ_f_*G*^0^(298 K), the curves corresponding to it have a quite distinct “zigzag” shape, because both of these parameters when passing from Ti to V, from Cr to Mn, from Fe to Ni, and decrease from Cu to Zn, but increase from V to Cr, from Mn to Fe, and Ni to Cu. However, the dynamics of the change of Δ_f_*S*^0^(298 K) in the Ti–Zn series are not similar to the dynamics of change as Δ_f_*H*^0^(298 K) and (Δ_f_*G*^0^(298 K), as well as the values of the temperature of the beginning of thermal destruction *T*_td_ [92,93]. It should be noted in this connection, that there is no correlation between the parameters of the atoms of the 3*d* elements that make up the Al_2_M_3_ metal clusters, which in principle can somehow be related to the thermodynamic characteristics (atomic numbers, atomic radii, ionization energies, etc.) [92,93] that are clearly visible in Figure 5 where the dependences of the thermodynamic parameters of the reaction (1) of 3*d*-element atomic number has been presented.

It should be noted that in the current literature there is also information on the molecular structures of some Al_2_M_3_ metal clusters, where M is a 4*d* element, namely Al_2_Mo_3_ and Al_2_Ag_3_, which is presented in the publications [84,85] and [90,91] cited above, respectively. However, this information is fragmentary and so far clearly insufficient for any serious generalizations.

## 4. Hexanuclear (AlM) Metal Clusters

**Figure 6:**
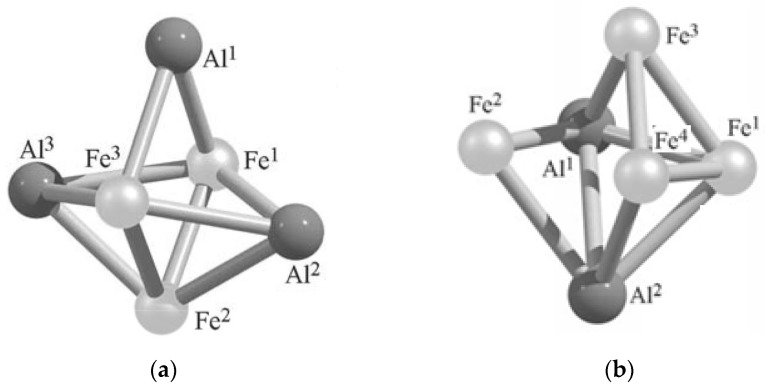
The images of molecular structures of most stable Al_3_Fe_3_ (**a**) and Al_2_Fe_4_ (**b**) isomers [95].

Theoretically, for metal clusters of such a category, the existence of five types of metal clusters is admissible, namely Al_5_M, Al_4_M_2_, Al_3_M_3_, Al_2_M_4_, and AlM_5,_ is possible. The situation here at the moment resembles that which occurs in the case of tetranuclear (AlM) metal clusters, since until now quantum-chemical calculations have been performed for only two types of such metal clusters containing the same chemical element, namely Al_3_Fe_3_ and Al_2_Fe_4_. The results of these calculations are presented in articles [74,75,94,95,96]. According to them, the first of these metal clusters can exist in 20 isomers [94,95,96], and the second in nine [95,96]. The relative total energies of all these isomers are presented in Table 8. In all isomers of the first of these metal cluster, direct Fe–Al valence bonds take place. In all of them, with the exception of only Al_3_Fe_3_ (XIII) and Al_3_Fe_3_ (XV), there are also at least one Fe–Fe and Al–Al bond. It is interesting to note in this connection that, according to the data of [94,95,96], namely the isomer of Al_3_Fe_3_ (XV), where Al–Al bonds are absent, that turns out to be the most stable in energy terms. The most important parameters of the molecular structure of this isomer are presented in Table 9, and its image in Figure 6a. However, the isomer Al_3_Fe_3_ (XIII), in which, on the contrary, there are no Fe–Fe bonds but there are Al–Al bonds, turns out to be one of the most high-energy isomers of the metal cluster under consideration (albeit not the most high-energy). Besides, the most energy-stable isomer of Al_3_Fe_3_ (XV) has a spin multiplicity of the ground state of 6, which corresponds to a high-spin state (Table 8). It is characteristic that the Al_3_Fe_3_ (XII) isomer which is nearest to it in energy, also has the same spin multiplicity, but its molecular structure differs significantly from the Al_3_Fe_3_ (XV) structure [94,95,96]. The following two structures with increasing energy, namely Al_3_Fe_3_ (V) and Al_3_Fe_3_ (XIV), have a lower spin multiplicity of 4. The most unstable isomer is Al_3_Fe_3_ (I), whose total energy is much higher (more than 150 kJ/mol) of that for the Al_3_Fe_3_ (XV) isomer and for which the spin multiplicity of the ground state is 2. Thus, it can be argued that, in general, for the isomers of Al_3_Fe_3_ metal cluster, the high-spin state is more characteristic.

The high-spin state, judging by the data of [95,96], is more characteristic in comparison with the low-spin state for Al_2_Fe_4_ clusters, too. In four of its 9 isomers, namely, Al_2_Fe_4_ (I), Al_2_Fe_4_ (II), Al_2_Fe_4_ (VIII), and Al_2_Fe_4_ (IX), there is a direct valence bond Al–Al, while in the other five isomers there is no such bond and there are only Fe–Fe and Fe–Al bonds in them. As can be seen from the data presented in Table 7, the most stable in energy terms is the isomer of Al_2_Fe_4_ (II), the image of the molecular structure of which is shown in Figure 6b, and its key parameters in Table 9. It is characteristic of the metal cluster under consideration that the most stable isomer of it also has the highest spin multiplicity, namely *M_S_* = 5. The isomers with a lower spin multiplicity equal to 3 generally have noticeably large total energies. At the same time, which is characteristic, among these isomers, there is not a single one the ground state of which would be a spin singlet. It is interesting that, according to the data of [95,96], in principle, an almost flat Al_2_Fe_4_ cluster with *M_S_* = 3 (and namely, Al_2_Fe_4_ (I) isomer) can exist, even though it is characterized by the highest total energy compared to other compounds of the same composition, and namely 165.5 kJ/mol **(Table 8)**. In this regard, it is worth noting that, according to the data of [87,88,89], flat structures are not found among five-atom Al_2_Fe_3_ metal clusters.

The images of molecular structures of all these hexanuclear metal clusters are presented in Supplementary Materials (Figures S13 and S14).

By highlighting, among other things, the general structural motifs of the isomers of hexa-nuclear (AlFe) metal clusters considered by us, we note that for most of these isomers, as well as for pentanuclear Al_2_M_3_, on the one hand, there are several metal–metal bonds formed by one and the same atom with its “neighbors”, on the other hand, relatively high values of the lengths of these bonds, which in all cases exceed 200 pm. From the average statistical point of view, the Al–Al bonds are the longest in full accordance with theoretical expectations, the Fe–Fe bonds are the shortest, while the Fe–Al bonds lengths occupy an intermediate position between the lengths of the bonds formed by two aluminum atoms and two iron atoms. It should be noted especially that all isomers of Al_3_Fe_3_ and Al_2_Fe_4_ metal clusters are either completely asymmetric, or have only one plane of symmetry. It is also characteristic that among the isomers of both Al_2_Fe_4_ and Al_3_Fe_3_ metal clusters, there is not one with a center of symmetry in its structure, although the structures of Al_2_Fe_4_ (I) and Al_2_Fe_4_ (V) are rather close to those [94,95,96].

## 5. Conclusions

As can be seen from the above literature data, tetranuclear (Al_2_M_2_), pentanuclear (Al_2_M_3_), and hexa-nuclear (Al_2_M_4_, Al_3_M_3_) metal clusters, containing at least two Al atoms and at least two M atoms of any of the 3*d* elements, form a rather significant number of structural isomers that differ significantly from each other in their structural and geometric parameters and in the values of the total energy. Moreover, most isomers of these metal clusters either do not have symmetry elements at all or have only one plane or one axis of symmetry. In many of these isomers, each of the aluminum atoms contained in their composition is connected by chemical bonds with three neighboring atoms, while for the atoms of the 3*d* element M, this feature, to all appearances, takes place to a lesser extent. Judging by the thermodynamic parameters of the reactions of their formation in the gas phase, the most studied (AlM) metal clusters, namely pentanuclear Al_2_M_3_ metal clusters, are capable of independent existence, and are very stable in thermal relation (i.e., to thermal destruction). At the same time, the standard thermodynamic characteristics of compounds of a given stoichiometric composition (Δ_f_*H*^0^(298 K), Δ_f_*S*^0^(298 K) and Δ_f_*G*^0^(298 K)) very strongly depend on the nature of the 3*d* element, and the dynamics of their change in a series Ti–Zn have a very complex and, moreover, a priori unpredictable character. Judging by the data presented in the articles published on this subject, no relationship between the total number of metal–metal bonds in the metal cluster and its relative total energy is also noted.

The (*pd*) metal clusters considered in the given paper, in the perspective, can be used primarily for creating new composite materials and alloys based on polymetallic nanoparticles. Other possible areas of application of these and similar chemical objects include the doping of traditional alloys based on both non-ferrous and ferrous metals, metal complex catalysis, the creation of specific electrochemical systems, and semiconductor technology. The data on the specifics of the molecular structures of the metal clusters examined by us, are quite capable of serving as a starting position on the basis of which it will be possible to calculate the structures of bicomponent (aluminum + (*d*-metal)) nanoclusters, which are associations of these metal clusters and contain from several tens to several hundred atoms. Furthermore, since most of the elementary processes associated with the formation of many chemical compounds, including the formation of metal structures and their alloys, occur in fragments of matter of a nanometer range with the above number of atoms, in the future, it will be possible to solve the problems of their influence on the physical chemical properties of polymetallic compositions. For example, to solve the question of whether an alloy, an intermetallic compound, or a mechanical mixture are formed when aluminum and *d*-metal are combined. It seems highly probable that, with their application as potential so-called quantum dots, the possibilities of this technology remain far from exhausted.

## Figures and Tables

**Table 1:** Relative energies and spin multiplicities of the ground states of various isomers of metal clusters Al_3_Fe, AlFe_3_ and Al_2_Fe_2_. Roman numerals in the table are the designations of these metal clusters presented in the original works.

Structure Designation	Spin Multiplicity of the Ground State	Relative Energy, kJ/mol	Ref.
**Al_3_Fe Metal Cluster**			
Al_3_Fe (I)	2	0.0	[79,80]
Al_3_Fe (VII)	2	175.1	
Al_3_Fe (II)	4	32.7	
Al_3_Fe (IV)	4	153.3	
Al_3_Fe (III)	6	83.4	
Al_3_Fe (VI)	6	84.8	
Al_3_Fe (V)	6	193.1	
**AlFe_3_ Metal Cluster**			
AlFe_3_ (II)	2	11.2	[79,80]
AlFe_3_ (I)	2	104.0	
AlFe_3_ (VII)	2	122.8	
AlFe_3_ (VIII)	2	198.7	
AlFe_3_ (V)	4	0.0	
AlFe_3_ (III)	4	11.3	
AlFe_3_ (IX)	4	150.2	
AlFe_3_ (VI)	6	17.4	
AlFe_3_ (IV)	6	41.7	
AlFe_3_ (X)	6	52.6	
**Al_2_Fe_2_ Metal Cluster**			
Al_2_Fe_2_ (XII)	1	45.1	[78,79,80]
Al_2_Fe_2_ (VII)	1	46.7	
Al_2_Fe_2_ (IX)	1	150.3	
Al_2_Fe_2_ (X)	1	209.8	
Al_2_Fe_2_ (III)	1	352.5	
Al_2_Fe_2_ (VI)	3	66.5	
Al_2_Fe_2_ (VIII)	3	68.4	
Al_2_Fe_2_ (IV)	3	137.9	
Al_2_Fe_2_ (XI)	3	143.6	
Al_2_Fe_2_ (II)	3	254.5	
Al_2_Fe_2_ (V)	5	0.0	
Al_2_Fe_2_ (I)	5	152.7	

**Table 2:** Key structural parameters of most stable Al_3_Fe, Al_2_Fe_2_ and AlFe_3_ metal clusters * [76,77,78,79,80].

Al_3_Fe Metal Cluster		Al_2_Fe_2_ Metal Cluster		AlFe_3_ Metal Cluster	
Metal-Metal Bond Lengths, pm		Metal-Metal Bond Lengths, pm		Metal-Metal Bond Lengths, pm	
Al1Al2	263.4	Al1Al2	260.8	Al1Fe1	249.1
Al1Al3	263.3	Al1Fe1	249.5	Al1Fe2	249.1
Al2Al3	274.3	Al1Fe2	249.4	Al1Fe3	255.5
Al1Fe1	245.7	Al2Fe1	249.5	Fe1Fe2	208.6
Al2Fe1	235.0	Al2Fe2	249.5	Fe1Fe3	*248.7*
Al3Fe1	235.1	Fe1Fe2	199.2	Fe2Fe3	*248.7*
**Bond Angles, deg**		**Bond Angles, deg**		**Bond Angles, deg**	
Al1Fe1Al2	66.4	Fe1Al1Fe2	47.1	Fe1Al1Fe2	49.5
Fe1Al1Al2	54.9	Fe1Al2Fe2	47.1	Al1Fe1Fe2	65.3
Al1Al2Fe1	58.7	Fe1Al1Al2	58.5	Fe1Fe2Al1	65.2
Al1Fe1Al3	66.4	Fe1Al2Al1	58.5	Fe1Al1Fe3	59.0
Al2Fe1Al3	71.4	Fe2Al1Al2	58.5	Fe2Al1Fe3	59.0
Al1Al2Al3	58.6	Fe2Al2Al1	58.5	Fe1Fe2Fe3	*65.2*
Al2Al3Al1	58.6	Al1Fe1Al2	63.0	Fe2Fe3Fe1	*49.6*
Al3Al1Al2	62.8	Al1Fe2Al2	63.1	Fe3Fe1Fe2	*65.2*

* The interatomic distances and angles within which at least one of the metal–metal bonds (Al–Al, Al–M, or M–M) is absent, are shown in this table in *italics*.

**Table 3:** Total number of Al_2_M_3_ (*N*) metal cluster isomers for various M of 3*d*-elements.

M	Ti	V	Cr	Mn	Fe	Co	Ni	Cu	Zn
*N*	14	11	20	25	8	9	7	8	14
Ref.	[81,83]	[82,83]	[84,85]	[86]	[87,88,89]	[87,88]	[87,88]	[89,90,91]	[86]

**Table 4:** Relative energies and spin multiplicities of the ground states of various isomers of 3*d*-element metal clusters having Al_2_M_3_ composition (M = Ti, V, Cr, Mn, Fe, Co, Ni, Cu, Zn). Roman numerals in the table are the designations of these metal clusters presented in the original works.

Structure Designation	Spin Multiplicity of the Ground State	Relative Energy, kJ/mol	Ref.
**Al_2_Ti_3_ Metal Cluster**			
Al_2_Ti_3_ (XIII)	1	21.5	[81,83]
Al_2_Ti_3_ (II)	1	24.1	
Al_2_Ti_3_ (VII)	1	44.8	
Al_2_Ti_3_ (XIV)	1	51.1	
Al_2_Ti_3_ (VIII)	1	77.5	
Al_2_Ti_3_ (III)	1	93.0	
Al_2_Ti_3_ (XII)	3	12.6	
Al_2_Ti_3_ (X)	3	37.0	
Al_2_Ti_3_ (VI)	3	37.2	
Al_2_Ti_3_ (I)	3	80.9	
Al_2_Ti_3_ (XI)	5	0.0	
Al_2_Ti_3_ (V)	5	19.7	
Al_2_Ti_3_ (IV)	5	60.7	
Al_2_Ti_3_ (IX)	5	73.0	
**Al_2_V_3_ Metal Cluster**			
Al_2_V_3_ (I)	2	25.9	[82,83]
Al_2_V_3_ (IV)	2	26.7	
Al_2_V_3_ (VII)	2	30.2	
Al_2_V_3_ (V)	4	0.0	
Al_2_V_3_ (II)	4	2.4	
Al_2_V_3_ (X)	4	59.6	
Al_2_V_3_ (VIII)	4	71.3	
Al_2_V_3_ (III)	6	18.8	
Al_2_V_3_ (VI)	6	26.8	
Al_2_V_3_ (XI)	6	74.6	
Al_2_V_3_ (IX)	6	141.0	
**Al_2_Cr_3_ Metal Cluster**			
Al_2_Cr_3_ (XVIII)	1	172.6	[84,85]
Al_2_Cr_3_ (XVI)	1	186.5	
Al_2_Cr_3_ (V)	1	197.2	
Al_2_Cr_3_ (XIII)	1	206.8	
Al_2_Cr_3_ (X)	1	219.0	
Al_2_Cr_3_ (XIX)	1	266.6	
Al_2_Cr_3_ (VI)	1	287.0	
Al_2_Cr_3_ (I)	1	396.2	
Al_2_Cr_3_ (VII)	3	79.4	
Al_2_Cr_3_ (II)	3	92.9	
Al_2_Cr_3_ (XIV)	3	109.3	
Al_2_Cr_3_ (XI)	3	109.6	
Al_2_Cr_3_ (III)	5	0.0	
Al_2_Cr_3_ (XVII)	5	13.1	
Al_2_Cr_3_ (VIII)	5	19.5	
Al_2_Cr_3_ (XII)	5	39.6	
Al_2_Cr_3_ (XX)	5	73.4	
Al_2_Cr_3_ (XV)	5	86.8	
Al_2_Cr_3_ (IV)	7	22.9	
Al_2_Cr_3_ (IX)	7	39.6	
**Al_2_Mn_3_ Metal Cluster**			
Al_2_Mn_3_ (XIII)	2	29.3	[86]
Al_2_Mn_3_ (XVII)	2	34.9	
Al_2_Mn_3_ (V)	2	47.5	
Al_2_Mn_3_ (XXII)	2	50.5	
Al_2_Mn_3_ (XXV)	2	80.8	
Al_2_Mn_3_ (VIII)	2	124.0	
Al_2_Mn_3_ (XIX)	2	129.4	
Al_2_Mn_3_ (XXI)	4	2.2	
Al_2_Mn_3_ (XII)	4	5.9	
Al_2_Mn_3_ (IV)	4	11.2	
Al_2_Mn_3_ (XVI)	4	27.0	
Al_2_Mn_3_ (VII)	4	29.6	
Al_2_Mn_3_ (XXIV)	4	76.1	
Al_2_Mn_3_ (II)	4	82.2	
Al_2_Mn_3_ (X)	4	141.7	
Al_2_Mn_3_ (VI)	6	0.0	
Al_2_Mn_3_ (XX)	6	1.4	
Al_2_Mn_3_ (III)	6	19.1	
Al_2_Mn_3_ (XV)	6	28.1	
Al_2_Mn_3_ (XI)	6	36.6	
Al_2_Mn_3_ (XIV)	6	42.3	
Al_2_Mn_3_ (XXIII)	6	62.4	
Al_2_Mn_3_ (IX)	6	74.6	
Al_2_Mn_3_ (I)	6	77.2	
Al_2_Mn_3_ (XVIII)	6	149.2	
**Al_2_Fe_3_ Metal Cluster**			
Al_2_Fe_3_(I)	1	273.7	[87,88,89]
Al_2_Fe_3_(IV)	1	300.6	
Al_2_Fe_3_(II)	3	0.0	
Al_2_Fe_3_(V)	3	24.1	
Al_2_Fe_3_(VII)	3	27.1	
Al_2_Fe_3_(III)	5	12.8	
Al_2_Fe_3_(VIII)	5	24.7	
Al_2_Fe_3_(VI)	5	31.1	
**Al_2_Co_3_ Metal Cluster**			
Al_2_Co_3_(I)	2	56.0	[87,88]
Al_2_Co_3_(VII)	2	83.6	
Al_2_Co_3_(IV)	2	92.0	
Al_2_Co_3_(II)	4	86.1	
Al_2_Co_3_(VIII)	4	86.8	
Al_2_Co_3_(V)	4	96.3	
Al_2_Co_3_(III)	6	0.0	
Al_2_Co_3_(IX)	6	28.9	
Al_2_Co_3_(VI)	6	73.3	
**Al_2_Ni_3_ Metal Cluster**			
Al_2_Ni_3_ (I)	1	48.6	[87,88]
Al_2_Ni_3_ (IV)	1	70.8	
Al_2_Ni_3_ (II)	3	0.0	
Al_2_Ni_3_ (V)	3	73.1	
Al_2_Ni_3_ (III)	5	102.6	
Al_2_Ni_3_ (VI)	5	113.3	
Al_2_Ni_3_ (VII)	5	148.4	
**Al_2_Cu_3_ Metal Cluster**			
Al_2_Cu_3_ (I)	2	0.0	[89,90,91]
Al_2_Cu_3_ (III)	2	20.6	
Al_2_Cu_3_ (V)	2	27.0	
Al_2_Cu_3_ (VII)	2	40.1	
Al_2_Cu_3_ (VIII)	2	67.6	
Al_2_Cu_3_ (IV)	4	136.9	
Al_2_Cu_3_ (II)	4	144.1	
Al_2_Cu_3_ (VI)	4	144.3	
**Al_2_Zn_3_ Metal Cluster**			
Al_2_Zn_3_ (III)	1	14.0	[86]
Al_2_Zn_3_ (II)	1	17.7	
Al_2_Zn_3_ (XII)	1	18.9	
Al_2_Zn_3_ (VI)	1	22.1	
Al_2_Zn_3_ (XIII)	1	23.8	
Al_2_Zn_3_ (XI)	1	25.6	
Al_2_Zn_3_ (V)	1	29.6	
Al_2_Zn_3_ (IX)	1	30.5	
Al_2_Zn_3_ (VIII)	1	79.2	
Al_2_Zn_3_ (I)	3	0.0	
Al_2_Zn_3_ (XIV)	3	3.2	
Al_2_Zn_3_ (IV)	3	11.0	
Al_2_Zn_3_ (VII)	3	21.4	
Al_2_Zn_3_ (X)	3	29.6	

**Table 5:** Key structural parameters of most stable Al_2_M_3_ clusters (M—3*d*-element) [92,93] *.

	M	Ti	V	Cr	Mn	Fe	Co	Ni	Cu	Zn
Parameter										
**Metal–Metal Bond Lengths, pm**										
Al1Al2		*422.7*	270.2	262.4	276.1	273.9	271.5	270.7	271.1	277.3
Al1M1		254.4	263.7	262.6	260.4	244.8	233.1	229.0	244.1	267.7
Al1M2		258.2	265.7	278.1	257.6	240.9	252.8	236.1	254.2	267.8
Al1M3		258.2	252.2	262.6	260.4	252.3	233.1	229.0	244.1	273.7
Al2M1		254.4	261.0	262.6	260.4	244.8	233.1	229.0	244.1	267.7
Al2M2		258.2	*378.8*	278.1	257.7	240.9	252.8	236.1	254.2	247.9
Al2M3		258.2	254.4	262.6	260.4	252.3	233.1	229.0	244.1	273.6
M1M3		258.7	*265.4*	*415.9*	*434.8*	*250.7*	*341.6*	*357.3*	*384.1*	*446.0*
M2M3		239.0	*253.3*	*258.1*	281.6	217.6	215.3	231.1	243.1	*446.1*
M1M2		258.8	171.7	*258.1*	281.6	*374.9*	215.3	231.1	243.1	247.9
**Bond Angles, deg**										
M1Al1M2		60.6	37.8	56.9	65.9	101.0	52.4	59.6	58.3	55.2
M1Al2M2		60.6	*22.9*	56.9	65.8	101.0	52.4	59.6	58.3	55.2
M1Al1Al2		*33.8*	58.5	60.0	58.0	56.0	54.4	53.8	56.3	58.8
M1Al2Al1		*33.8*	59.5	60.0	58.0	56.0	54.4	53.8	56.3	58.8
M2Al1Al2		*35.6*	90.0	61.9	57.6	55.4	57.5	55.0	57.8	58.8
M2Al2Al1		*35.0*	*44.5*	61.9	57.6	55.4	57.5	55.0	57.8	58.8
Al1M1Al2		112.3	62.0	59.9	64.0	68.0	71.2	72.5	67.4	62.4
Al1M2Al2		109.9	*45.5*	56.3	64.8	69.3	64.9	69.9	64.4	62.4
Al1M3Al2		109.9	64.5	59.9	64.0	65.7	68.5	72.5	67.4	60.9
M1Al1M3		60.6	*61.9*	104.7	113.2	60.5	94.2	102.6	103.7	110.9
M1Al2M3		60.6	62.0	104.7	113.2	60.5	94.2	102.6	103.7	111.0
M1M3M2		62.5	*38.6*	*36.3*	65.9	*106.2*	*37.5*	*39.4*	*37.8*	*32.3*
M2Al1M3		55.1	58.5	56.9	65.9	52.3	52.4	59.6	58.3	110.9
M2Al2M3		55.1	*41.6*	56.9	101.1	52.3	52.4	59.6	58.3	111.0
M1M2M3		62.5	*74.5*	*107.3*	*39.5*	40.0	105.0	101.3	*104.4*	*73.9*

* The interatomic distances and angles within which at least one of the metal–metal bonds (Al–Al, Al–M, or M–M) is absent, are shown in this table in *italics*.

**Table 6:** Standard thermodynamic parameters of formation for the most energy-stable Al_2_M_3_ metal clusters and the reactions of their formation from atoms of the chemical elements constituting them, in the gas phase [92,93].

Metal Cluster	Standard Thermodynamic Parameters of Formation		
	Δ_f_*H*^0^(298 K) kJ/mol	Δ_f_*S*^0^(298 K) J/mol∙ K	Δ_f_*G*^0^(298 K) kJ/mol
Al_2_Ti_3_	967.4	429.9	883.5
Al_2_V_3_	526.5	438.5	433.8
Al_2_Cr_3_	1151.1	417.8	1067.5
Al_2_Mn_3_	516.8	423.1	436.2
Al_2_Fe_3_	823.4	430.0	736.5
Al_2_Co_3_	817.6	427.6	733.9
Al_2_Ni_3_	760.9	430.0	676.3
Al_2_Cu_3_	812.2	406.9	737.7
Al_2_Zn_3_	700.1	445.1	621.7
**Metal Cluster**	**Standard Thermodynamic Parameters of Reactions 2Al(gas) + 3M(gas)→Al_2_M_3_ (gas)**		
	**Δ*H*_298_, kJ/mol**	**Δ*S*_298_, J/mol∙ K**	**Δ*G*_298_, kJ/mol**
Al_2_Ti_3_	–1098.5	–439.5	–813.4
Al_2_V_3_	–1672.0	–436.9	–1392.1
Al_2_Cr_3_	–694.0	–433.6	407.7
Al_2_Mn_3_	–973.4	–426.5	–692.2
Al_2_Fe_3_	–1078.1	–440.0	–793.0
Al_2_Co_3_	–1109.1	–439.4	–824.1
Al_2_Ni_3_	–1162.8	–445.1	–876.0
Al_2_Cu_3_	–858.3	–420.8	–578.8
Al_2_Zn_3_	–344.6	–366.4	–84.3

**Table 7:** Thermal destruction onset temperatures (*Ttd*) for energetically most stable Al2M3 metal clusters [92,93].

Metal Cluster	Atomic Number of M	*T*_td_, K
Al_2_Ti_3_	22	2502.2
Al_2_V_3_	23	3826.0
Al_2_Cr_3_	24	1599.0
Al_2_Mn_3_	25	2278.6
Al_2_Fe_3_	26	2450.2
Al_2_Co_3_	27	2526.4
Al_2_Ni_3_	28	2613.0
Al_2_Cu_3_	29	2038.7
Al_2_Zn_3_	30	941.5

**Table 8:** Relative energies and spin multiplicities of the ground states of various isomers of metal clusters Al_3_Fe_3_ and Al_2_Fe_4_. Roman numerals in the table are the designations of these metal clusters presented in the original works.

Structure Designation	Spin Multiplicity of the Ground State	Relative Energy, kJ/mol	Ref.
**Al_3_Fe_3_ Metal Cluster**			
Al_3_Fe_3_ (IV)	2	58.8	[94,95,96]
Al_3_Fe_3_ (X)	2	95.2	
Al_3_Fe_3_ (XIX)	2	108.3	
Al_3_Fe_3_ (VII)	2	134.5	
Al_3_Fe_3_ (XIII)	2	137.2	
Al_3_Fe_3_ (XVI)	2	153.7	
Al_3_Fe_3_ (I)	2	158.3	
Al_3_Fe_3_ (V)	4	56.8	
Al_3_Fe_3_ (XIV)	4	56.8	
Al_3_Fe_3_ (VIII)	4	108.9	
Al_3_Fe_3_ (XI)	4	110.1	
Al_3_Fe_3_ (II)	4	130.8	
Al_3_Fe_3_ (XV)	6	0.0	
Al_3_Fe_3_ (XII)	6	40.8	
Al_3_Fe_3_ (IX)	6	71.8	
Al_3_Fe_3_ (VI)	6	77.3	
Al_3_Fe_3_ (III)	6	78.1	
Al_3_Fe_3_ (XX)	6	105.9	
Al_3_Fe_3_ (XVII)	6	111.4	
Al_3_Fe_3_ (XVIII)	6	155.4	
**Al_2_Fe_4_ Metal Cluster**			
Al_2_Fe_4_ (V)	3	13.0	[95,96]
Al_2_Fe_4_ (VIII)	3	27.5	
Al_2_Fe_4_ (III)	3	80.8	
Al_2_Fe_4_ (IX)	3	102.8	
Al_2_Fe_4_ (VI)	3	115.7	
Al_2_Fe_4_ (I)	3	165.5	
Al_2_Fe_4_ (II)	5	0.0	
Al_2_Fe_4_ (VII)	5	25.5	
Al_2_Fe_4_ (IV)	5	79.2	

**Table 9:** Key structural parameters of most stable Al_3_Fe_3_ and Al_2_Fe_4_ metal clusters * [94,95,96].

Al_3_Fe_3_ Metal Cluster		Al_2_Fe_4_ Metal Cluster	
Metal–Metal Bond Lengths, pm		Metal–Metal Bond Lengths, pm	
Al1Al2	*306.2*	Al1Fe1	242.7
Al1Al3	*307.1*	Al1Fe2	249.8
Al2Al3	*398.9*	Al1Fe3	243.8
Al1Fe1	236.4	Al1Fe4	*350.4*
Al1Fe2	*370.1*	Al1Al2	274.7
Al1Fe3	236.3	Al2Fe1	242.7
Al2Fe1	244.4	Al2Fe2	249.7
Al2Fe2	242.1	Al2Fe3	*350.4*
Al2Fe3	244.2	Al2Fe4	243.8
Al3Fe1	244.3	Fe2Fe3	*244.1*
Al3Fe2	242.1	Fe1Fe4	225.9
Al3Fe3	244.2	Fe1Fe3	225.9
Fe1Fe2	222.5	Fe1Fe2	*329.1*
Fe1Fe3	*270.9*	Fe2Fe4	244.1
Fe2Fe3	222.4	Fe3Fe4	230.7
**Bond Angles, deg**		**Bond Angles, deg**	
Fe2Al1Fe3	*35.0*	Fe2Al1Fe3	59.3
Fe1Al1Fe2	*35.0*	Fe1Al1Fe2	83.9
Fe1Al2Al3	*35.3*	Fe1Al2Fe4	55.4
Fe2Al2Al3	*34.5*	Fe2Al2Fe4	59.3
Al1Al2Fe2	*84.1*	Al1Al2Fe2	56.6
Fe2Al1Al2	*40.6*	Fe2Al1Al2	56.6
Al1Al2Al3	*49.5*	Al1Al2Fe4	84.8
Al2Al3Fe1	*35.3*	Al2Fe4Fe1	62.1
Al3Fe1Al2	109.4	Fe4Fe1Al2	62.6
Fe3Al1Al2	*51.6*	Fe3Al1Al2	84.8
Fe1Al1Fe3	69.9	Fe1Al1Fe3	55.4
Fe1Al1Al2	*51.6*	Fe1Al1Al2	55.5
Al1Fe1Al2	79.1	Al1Fe1Al2	68.9
Al3Fe2Fe3	63.3	Fe4Fe2Fe3	56.4

* The interatomic distances and angles within which at least one of the metal–metal bonds (Al–Al, Al–Fe, or Fe–Fe) is absent, are shown in this table *in italics*.

## Supplementary Materials

The following are available online at https://www.mdpi.com/1996-1944/13/8/1852/s1, Figure S1: Molecular structures of Al_3_Fe clusters, Figure S2: Molecular structures of Al_2_Fe_2_ clusters, Figure S3: Molecular structures of AlFe_3_ clusters, Figure S4: Molecular structures of Al_2_Ti_3_ clusters, Figure S5: Molecular structures of Al_2_V_3_ clusters, Figure S6: Molecular structures of Al_2_Cr_3_ clusters, Figure S7: Molecular structures of Al_2_Mn_3_ clusters, Figure S8: Molecular structures of Al_2_Fe_3_ clusters, Figure S9: Molecular structures of Al_2_Co_3_ clusters, Figure S10: Molecular structures of Al_2_Ni_3_ clusters, Figure S11: Molecular structures of Al_2_Cu_3_ clusters, Figure S12: Molecular structures of Al_2_Zn_3_ clusters, Figure S13: Molecular structures of Al_3_Fe_3_ clusters, Figure S14: Molecular structures of Al_2_Fe_4_ clusters.
