# Supplementary material for: Quantum-Chemical Design of Molecular Structures of Tetra-, Penta- and Hexanuclear Metal Clusters Containing Aluminum and 3d-Element Atoms
Source: Materials (Basel). 2020 Apr 15;13(8):1852. doi: 10.3390/ma13081852 (PMC7215831; doi:10.3390/ma13081852)
Supplement: Supplementary file 1 [file materials-13-01852-s001.pdf]

Supplemental Materials Title

Quantum-Chemical Design of Molecular Structures of Tetra-, Penta- and Hexanuclear Metal Clusters Containing Aluminum and 3*d*-Element Atoms

Oleg V. Mikhailov [1,*] and Denis V. Chachkov [2]

[1] Department of Analytical Chemistry, Certification and Quality Management, Kazan National Research Technological University, K. Marx Street 68, 420015 Kazan, Russia

[2] Kazan Department of Joint Supercomputer Center of Russian Academy of Sciences – Branch of Federal Scientific Center "Scientific Research Institute for System Analysis of the RAS", Lobachevskii Street 2/31, 420111 Kazan, Russia; de2005c@gmail.com

* Correspondence: olegmkhlv@gmail.com

Received: 07 March 2020; Accepted: 13 April 2020; Published: date.

Molecular Structures of Tetranuclear Al_3M, Al_2M_2 and AlM_3 metal clusters (M = Fe)

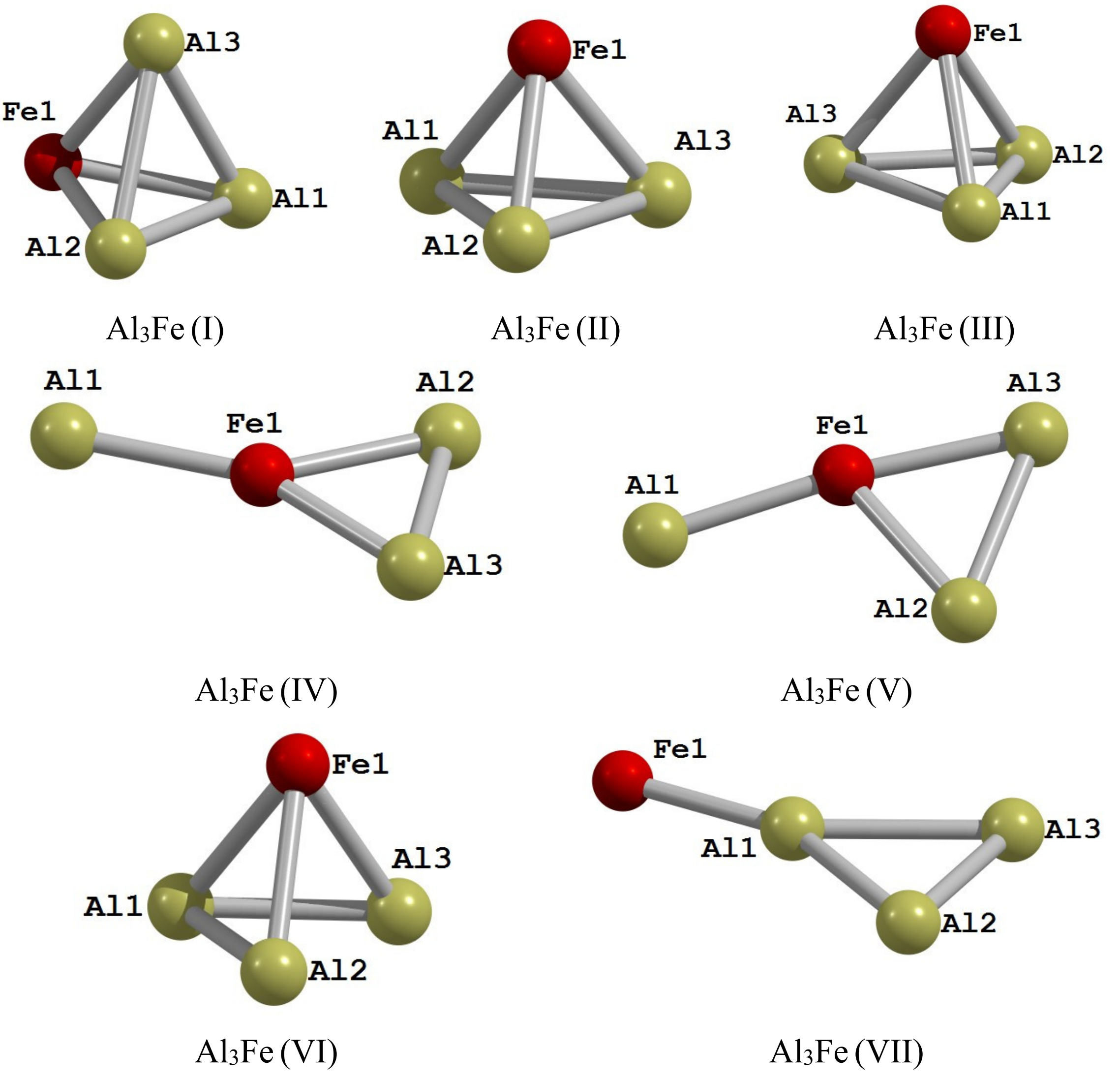

Figure S1. Molecular structures of Al_3Fe clusters.

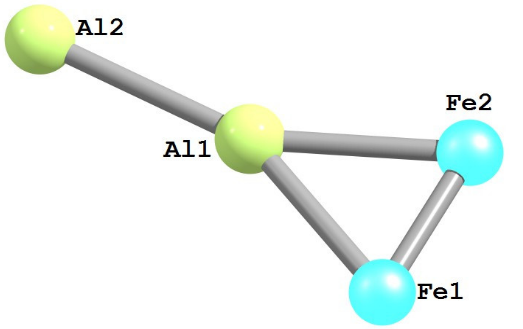

Al_2Fe_2 (I)

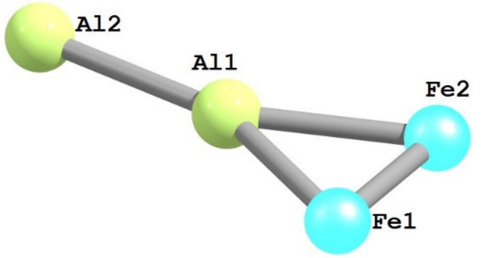

Al_2Fe_2 (II)

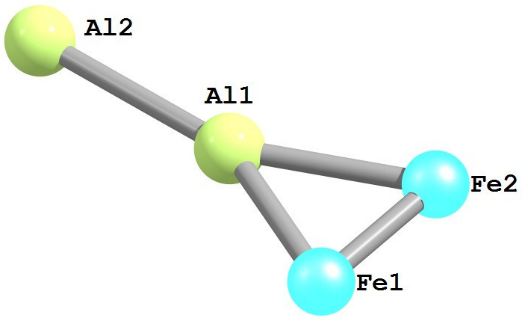

Al_2Fe_2 (III)

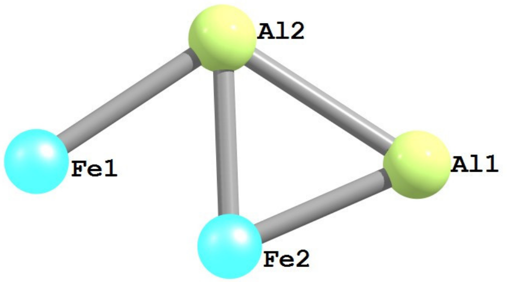

Al_2Fe_2 (IV)

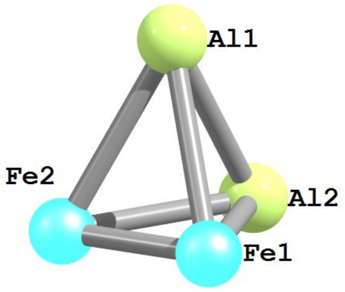

Al_2Fe_2 (V)

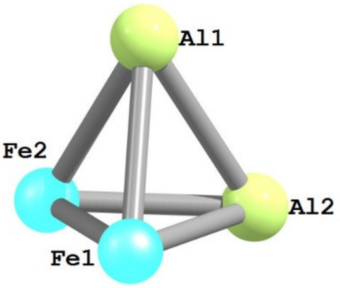

Al_2Fe_2 (VI)

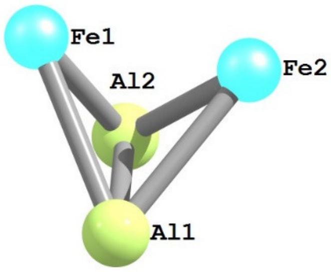

Al_2Fe_2 (VII)

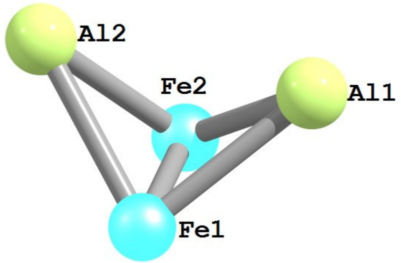

Al_2Fe_2 (VIII)

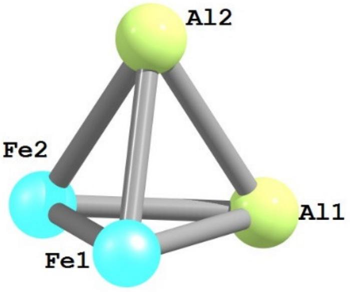

Al_2Fe_2 (IX)

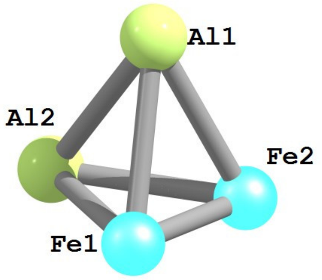

Al_2Fe_2 (X)

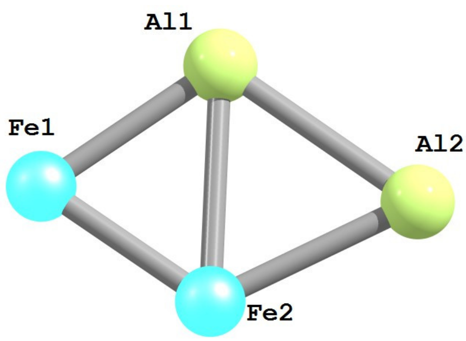

Al_2Fe_2 (XI)

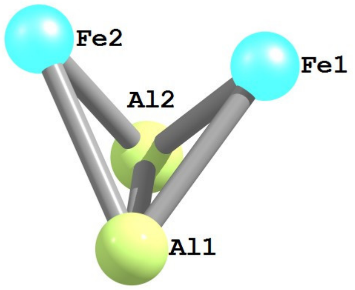

Al_2Fe_2 (XII)

Figure S2. Molecular structures of Al_2Fe_2 clusters.

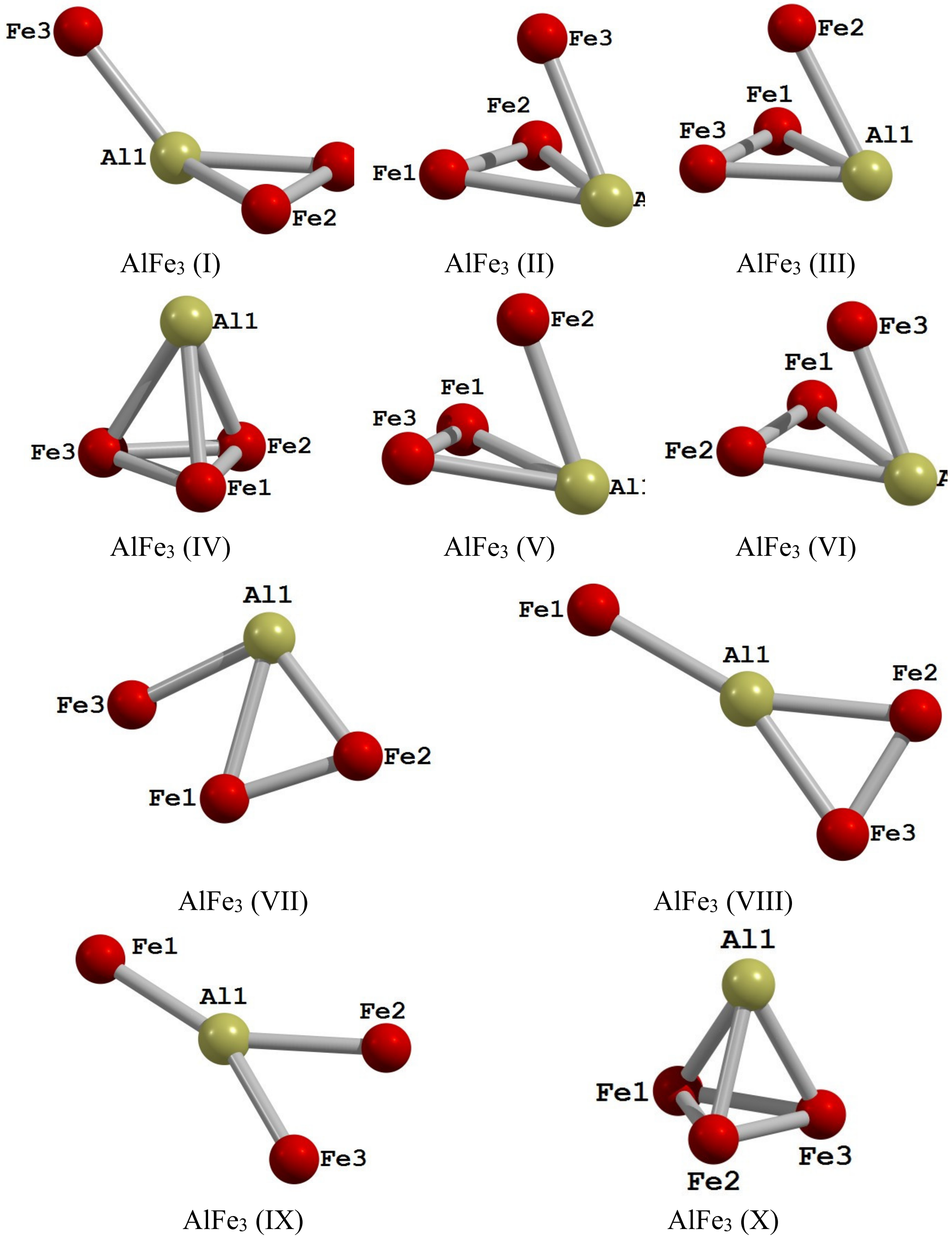

Figure S3. Molecular structures of $AlFe_3$ clusters.

Molecular Structures of Al_2M_3 Metal Clusters (M- 3*d*-element)

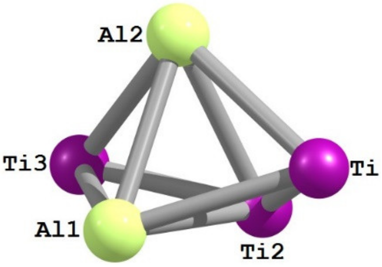

Al_2Ti_3 (I)

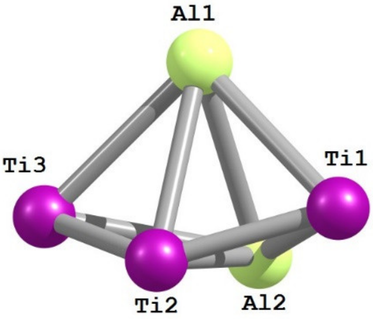

Al_2Ti_3 (II)

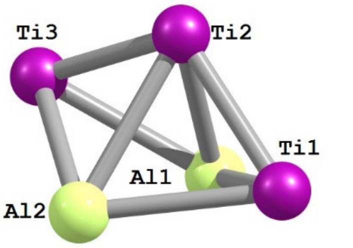

Al_2Ti_3 (III)

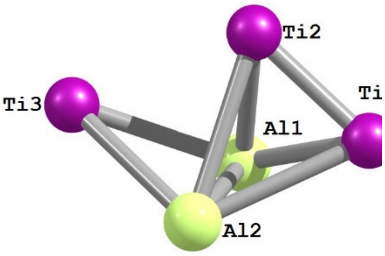

Al_2Ti_3 (IV)

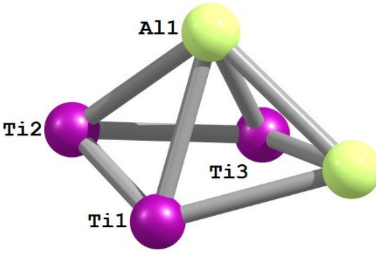

Al_2Ti_3 (V)

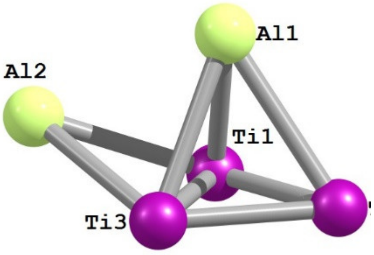

Al_2Ti_3 (VI)

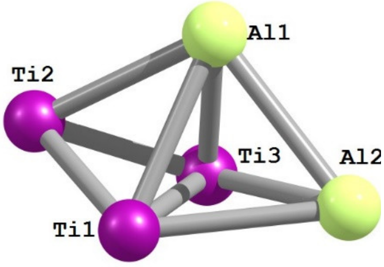

Al_2Ti_3 (VII)

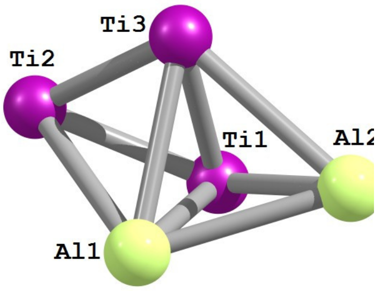

Al_2Ti_3 (VIII)

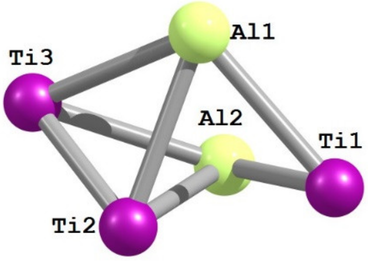

Al_2Ti_3 (IX)

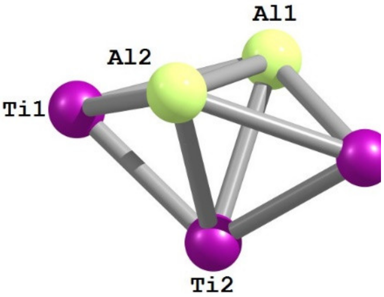

Al_2Ti_3 (X)

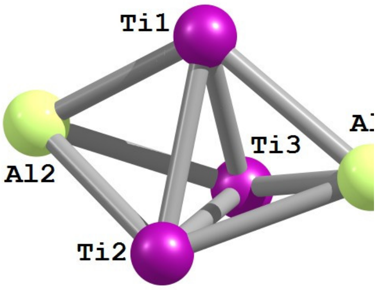

Al_2Ti_3 (XI)

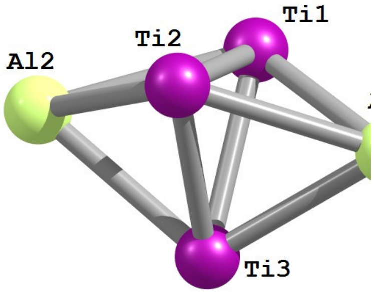

Al_2Ti_3 (XII)

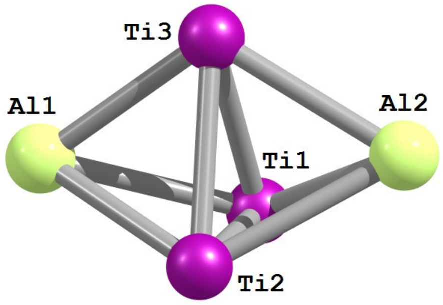

Al_2Ti_3 (XIII)

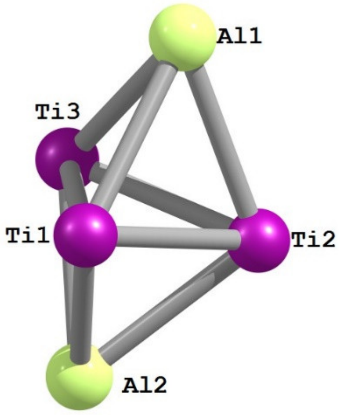

Al_2Ti_3 (XIV)

Figure S4. Molecular structures of Al_2Ti_3 clusters.

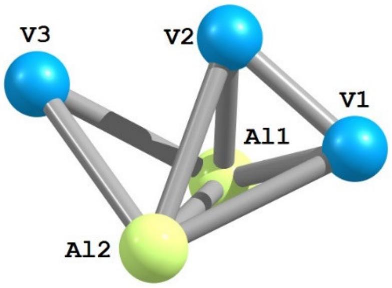

Al_2V_3 (I)

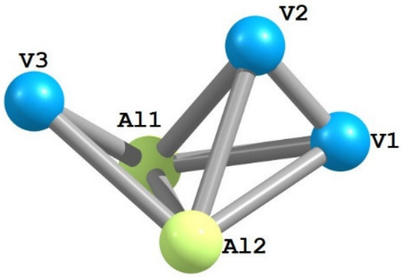

Al_2V_3 (II)

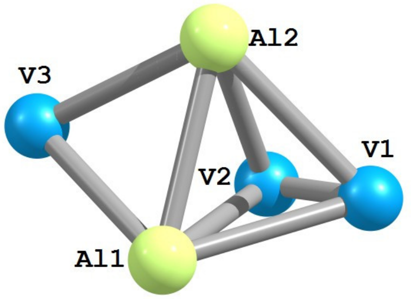

Al_2V_3 (III)

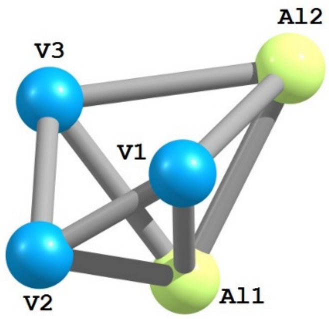

Al_2V_3 (IV)

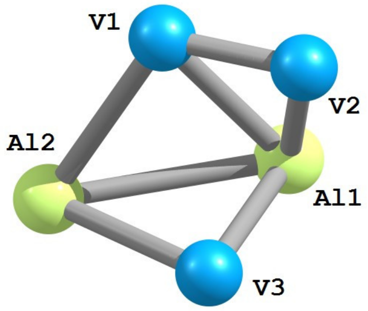

Al_2V_3 (V)

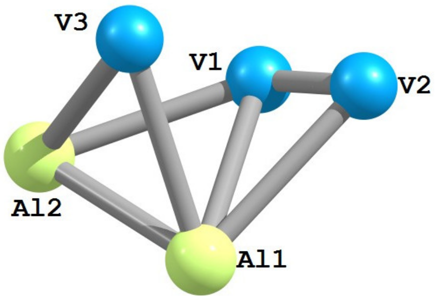

Al_2V_3 (VI)

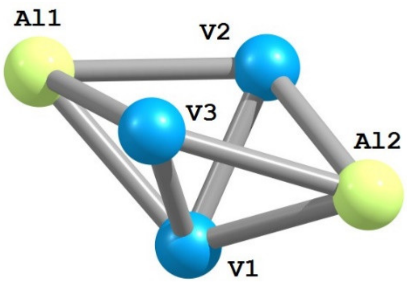

Al_2V_3 (VII)

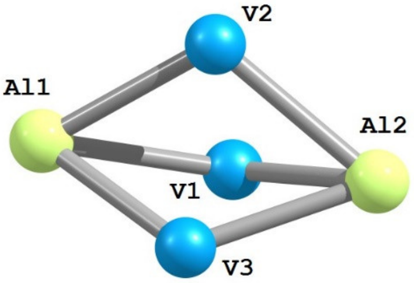

Al_2V_3 (VIII)

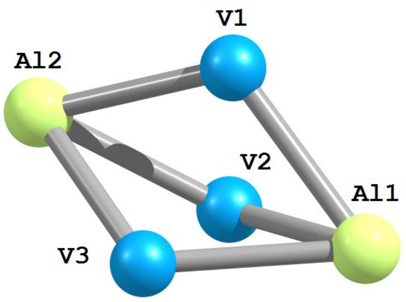

Al_2V_3 (IX)

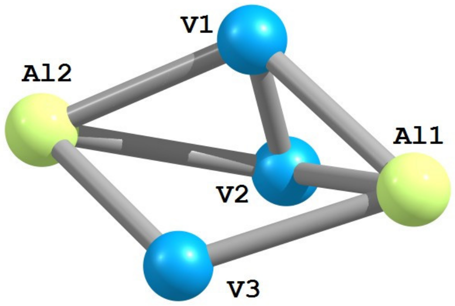

Al_2V_3 (X)

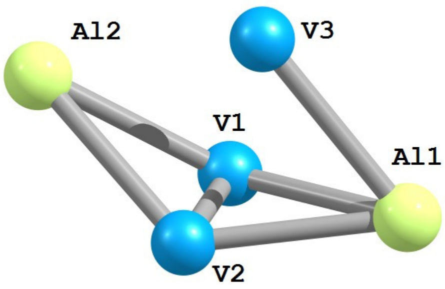

Al_2V_3 (XI)

Figure S5. Molecular structures of Al_2V_3 clusters.

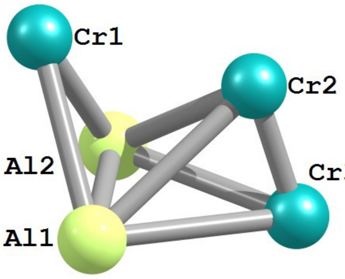

Al_2Cr_3 (I)

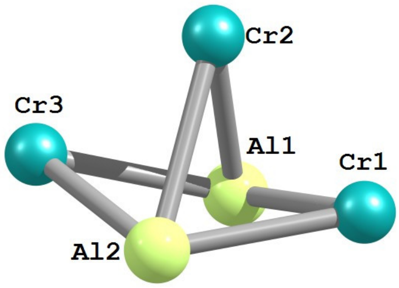

Al_2Cr_3 (II)

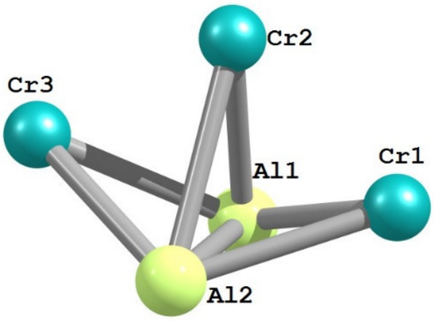

Al_2Cr_3 (III)

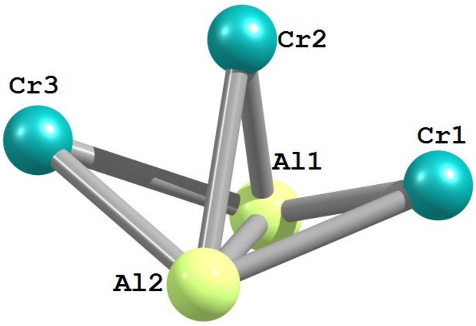

Al_2Cr_3 (IV)

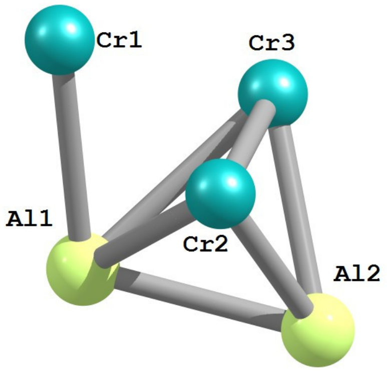

Al_2Cr_3 (V)

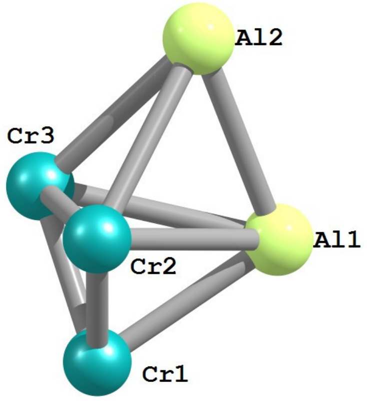

Al_2Cr_3 (VI)

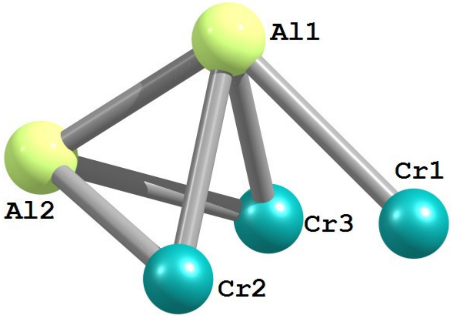

Al_2Cr_3 (VII)

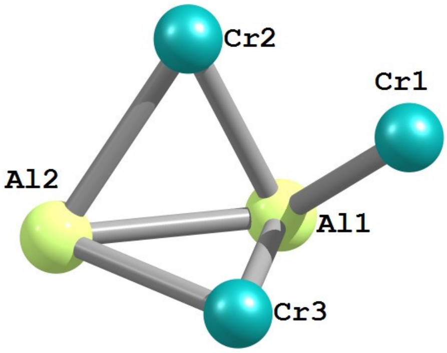

Al_2Cr_3 (VIII)

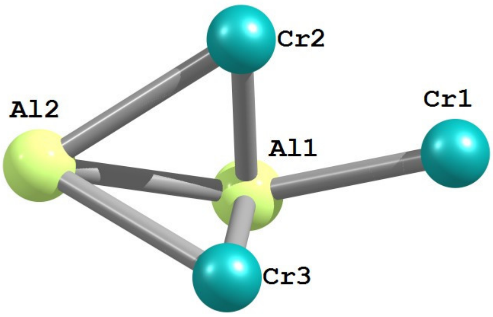

Al_2Cr_3 (IX)

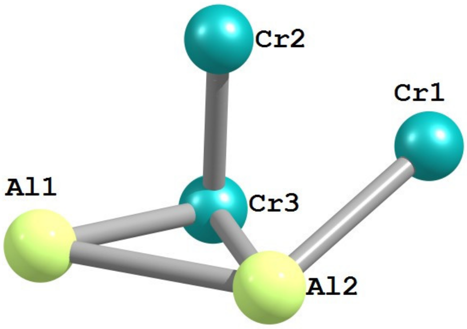

Al_2Cr_3 (X)

Figure S6. (a) Molecular structures of Al_2Cr_3 (I) – Al_2Cr_3 (X) clusters.

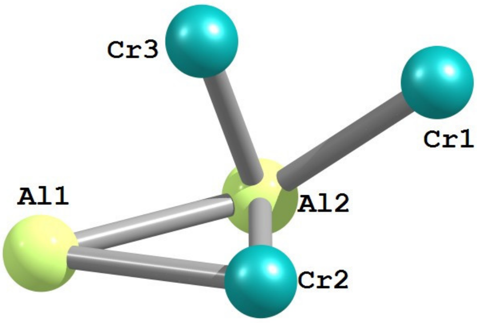

Al_2Cr_3 (XI)

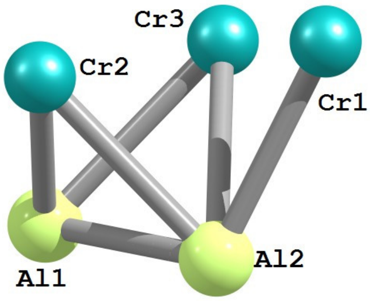

Al_2Cr_3 (XII)

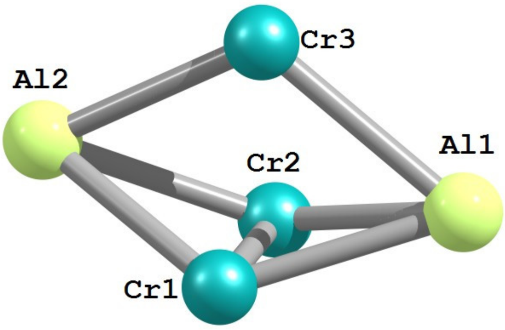

Al_2Cr_3 (XIII)

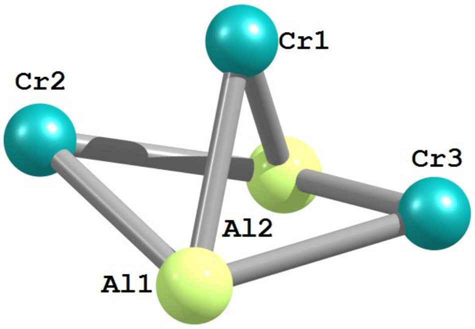

Al_2Cr_3

(XIV)

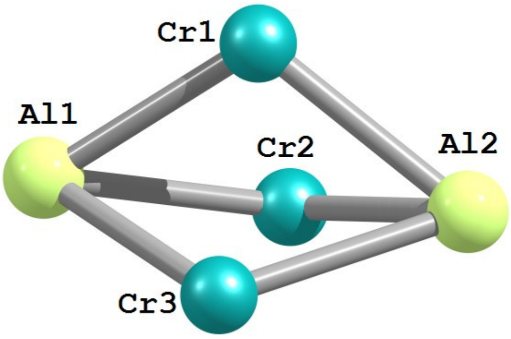

Al_2Cr_3 (XV)

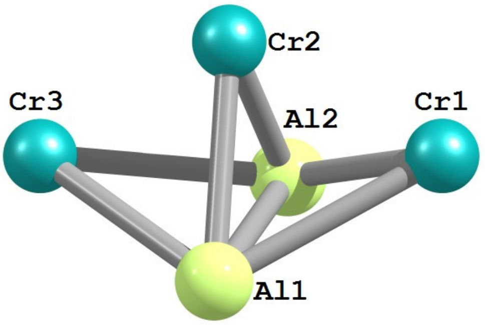

Al_2Cr_3

(XVI)

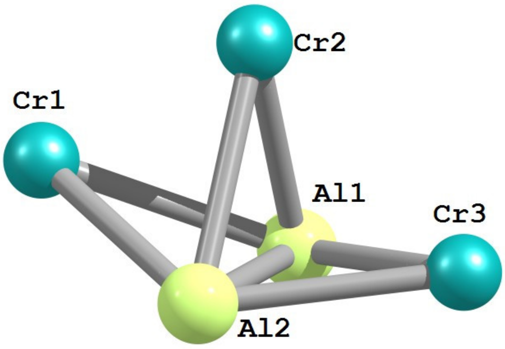

Al_2Cr_3 (XVII)

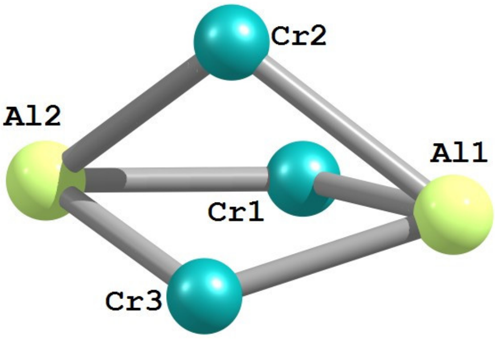

Al_2Cr_3

(XVIII)

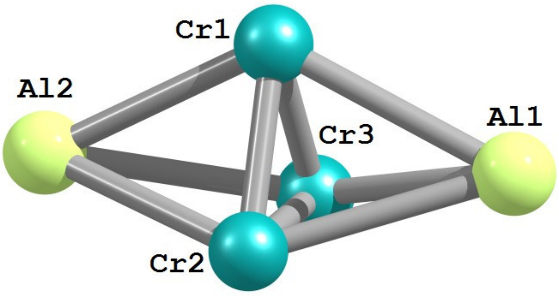

Al_2Cr_3 (XIX)

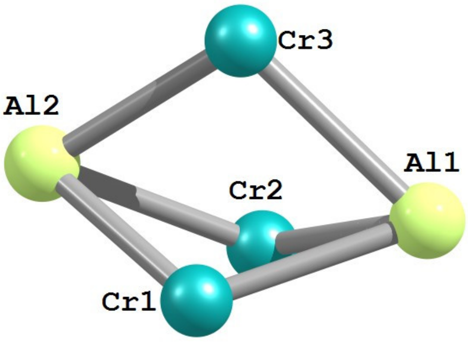

Al_2Cr_3 (XX)

Figure 6. (**b**) Molecular structures of Al_2Cr_3 (XI) – Al_2Cr_3 (XX) clusters.

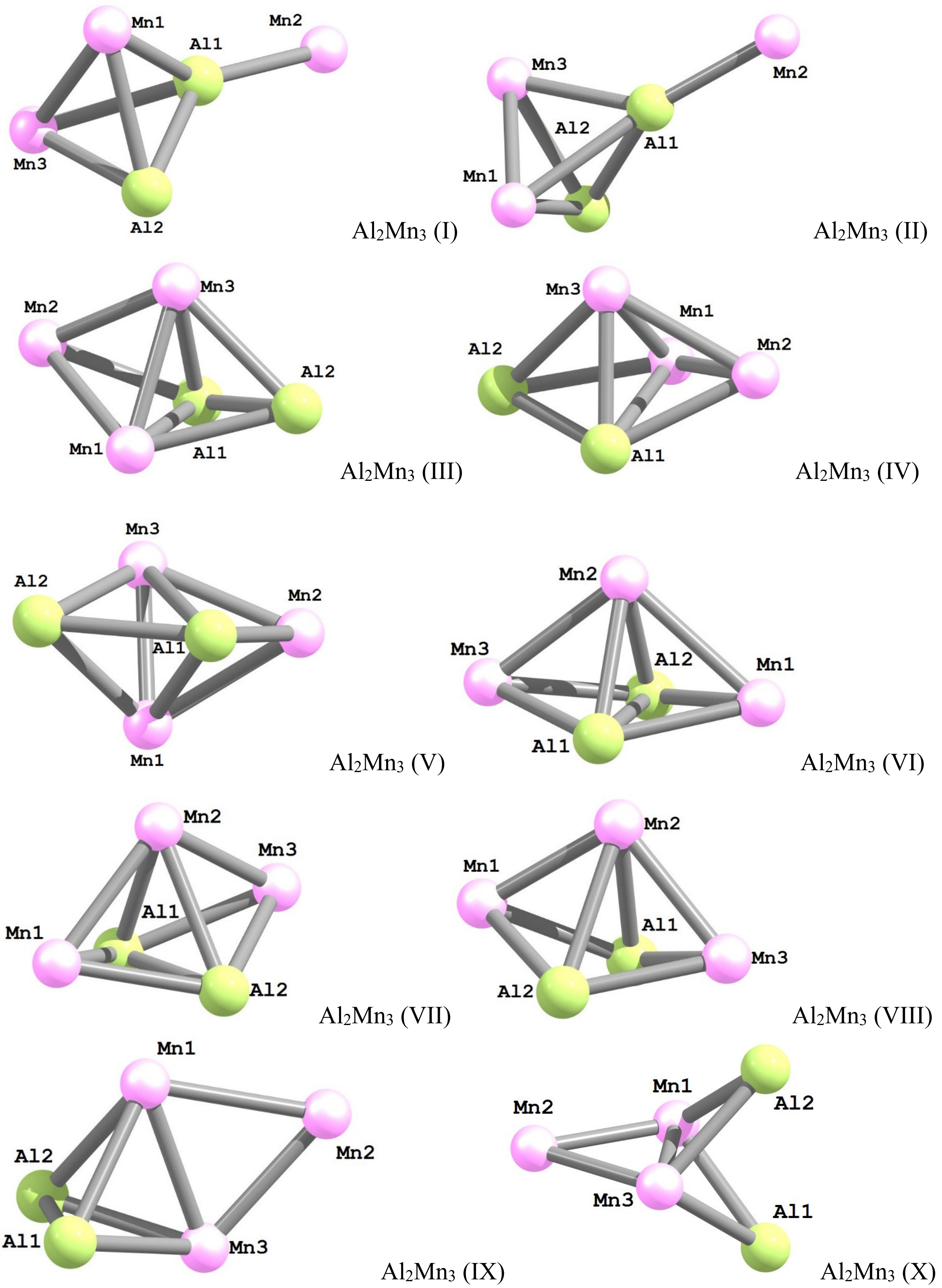

Figure 7. (a). Molecular structures of Al_2Mn_3 (I) – Al_2Mn_3 (X) clusters.

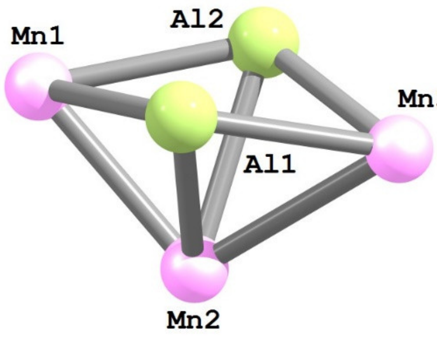

Al_2Mn_3 (XI)

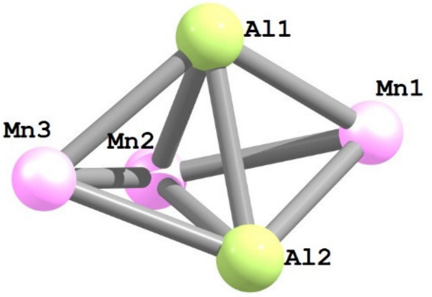

Al_2Mn_3 (XII)

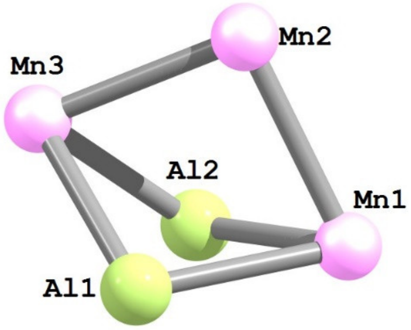

Al_2Mn_3 (XIII)

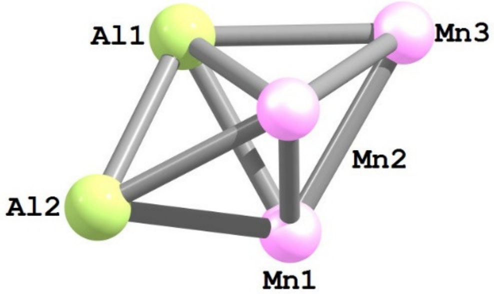

Al_2Mn_3 (XIV)

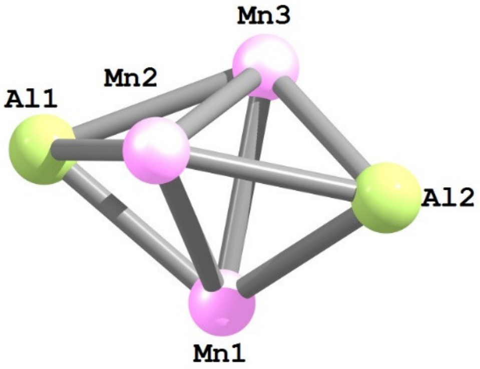

Al_2Mn_3 (XV)

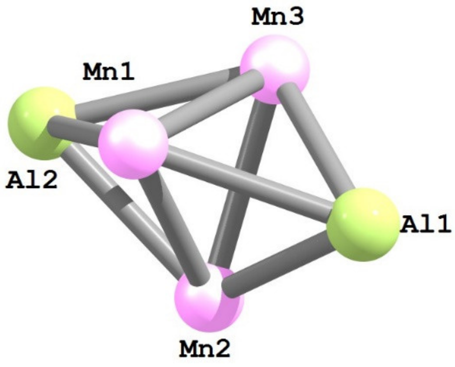

Al_2Mn_3 (XVI)

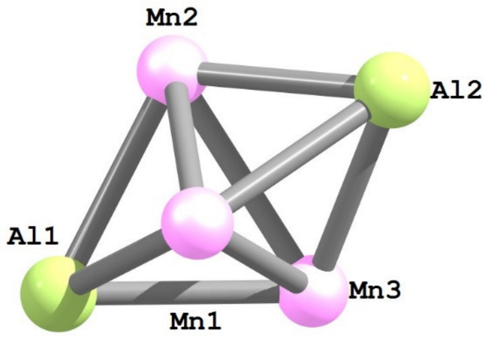

Al_2Mn_3 (XVII)

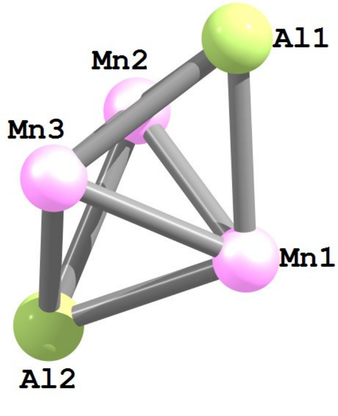

Al_2Mn_3

(XVIII)

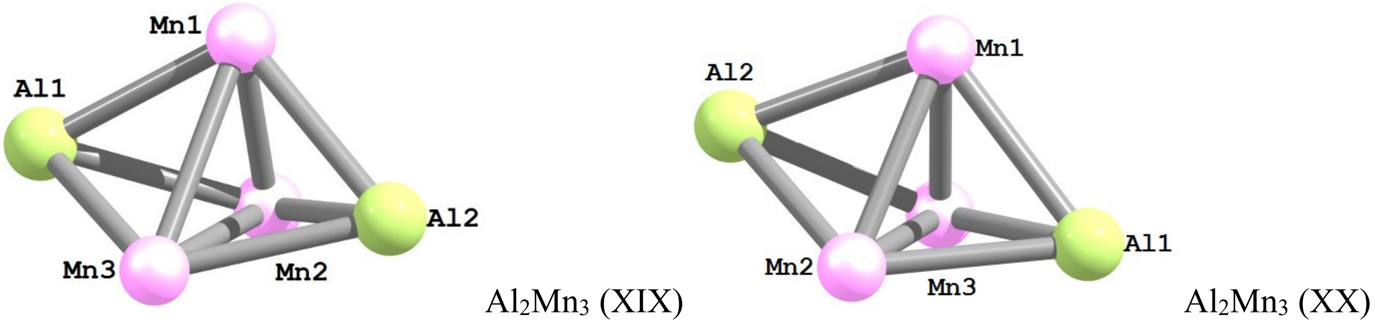

Figure 7. (b). Molecular structures of Al_2Mn_3 (XI) – Al_2Mn_3 (XX) clusters.

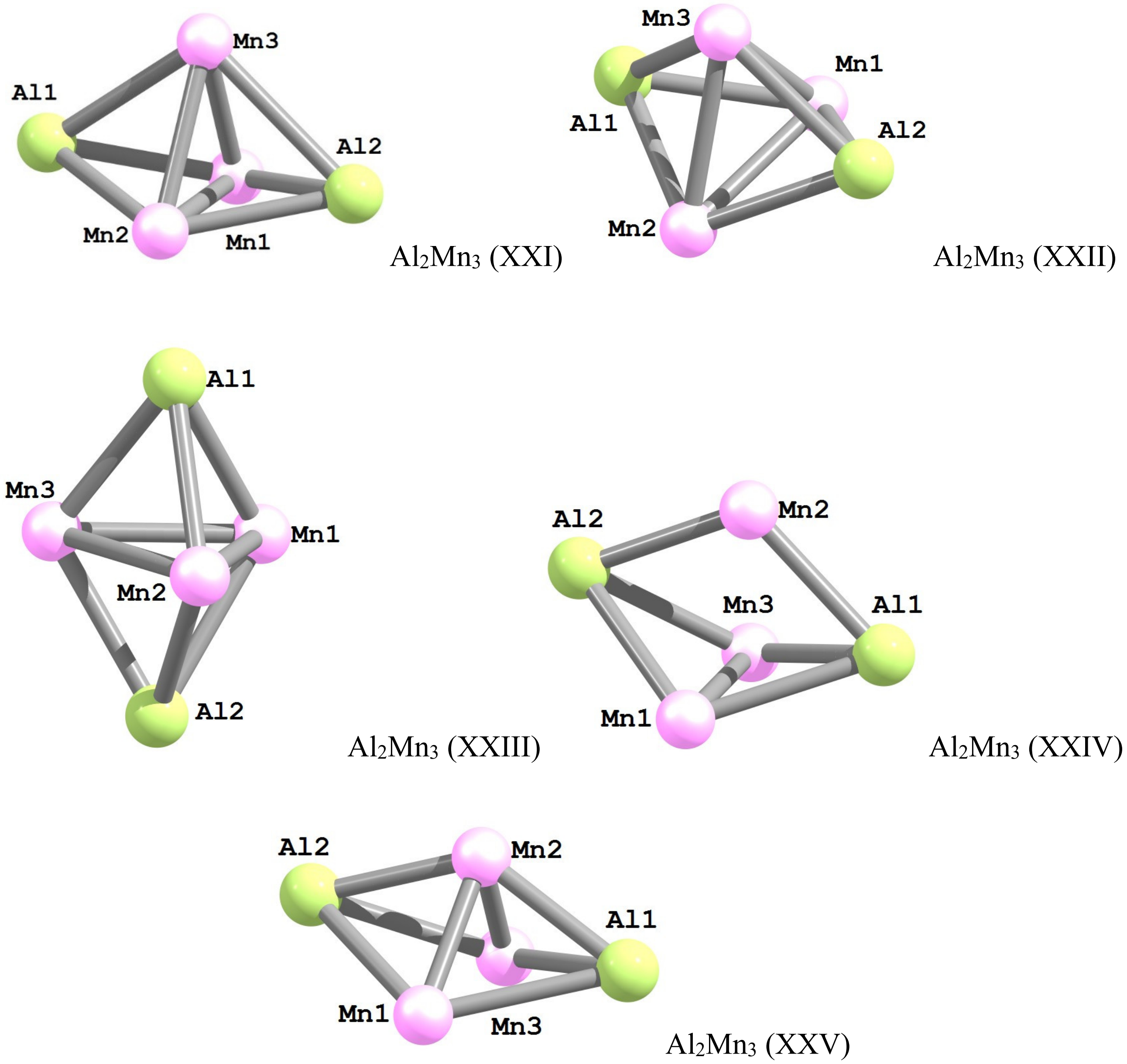

Figure 7. (c). Molecular structures of Al_2Mn_3 (XXI) – Al_2Mn_3 (XXV) clusters.

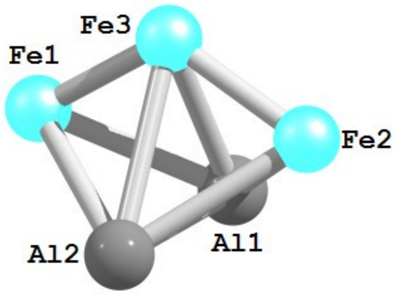

Al_2Fe_3 (I)

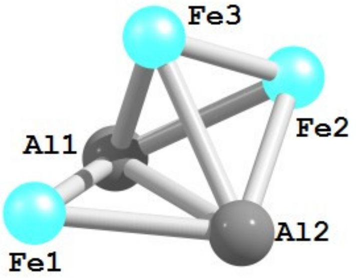

Al_2Fe_3 (II)

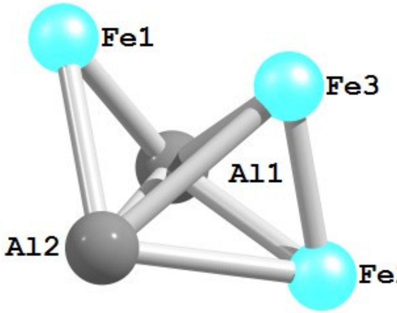

Al_2Fe_3 (III)

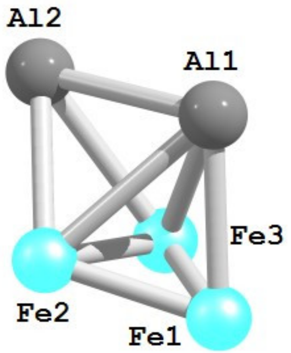

Al_2Fe_3 (IV)

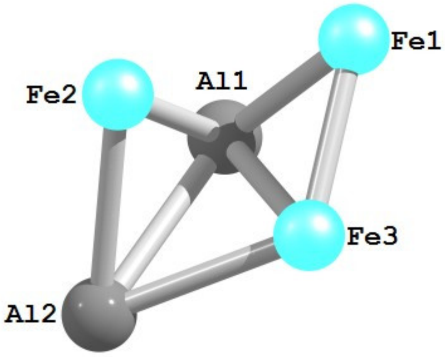

Al_2Fe_3 (V)

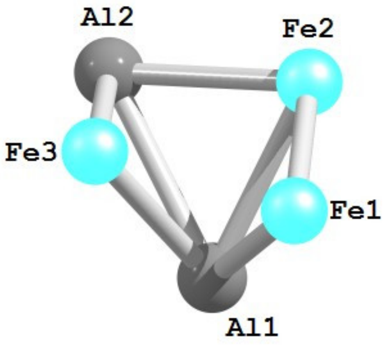

Al_2Fe_3 (VI)

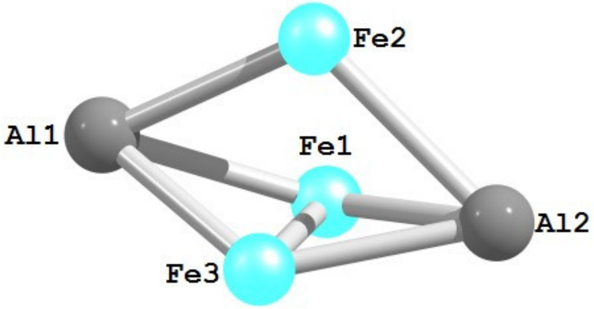

Al_2Fe_3(VII)

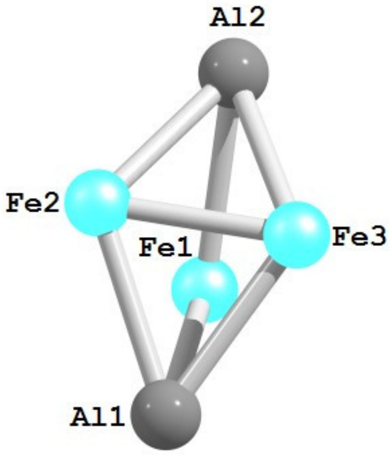

Al_2Fe_3(VIII)

Figure S8. Molecular structures of Al_2Fe_3 clusters.

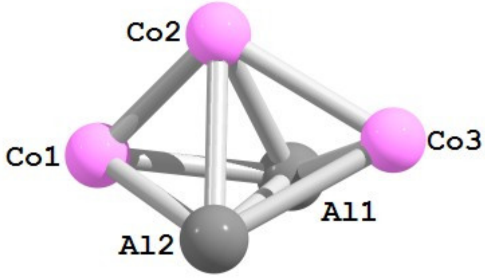

Al_2Co_3 (I)

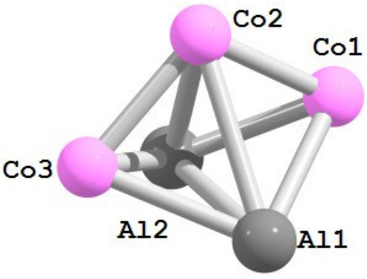

Al_2Co_3 (II)

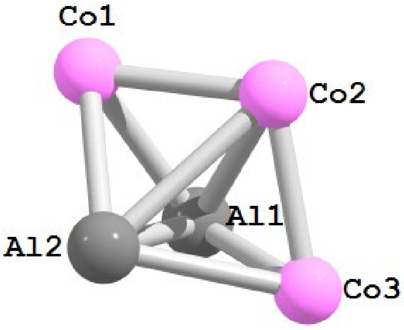

Al_2Co_3 (III)

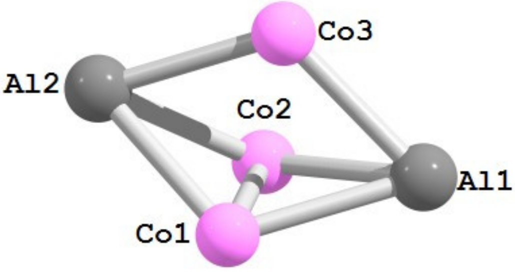

Al_2Co_3 (IV)

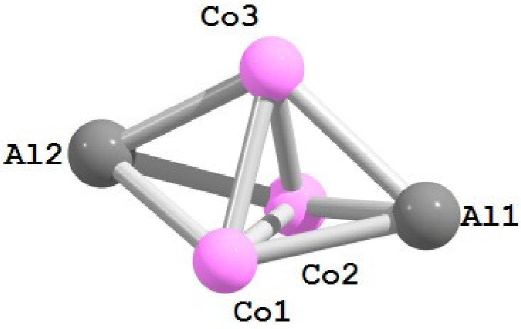

Al_2Co_3 (V)

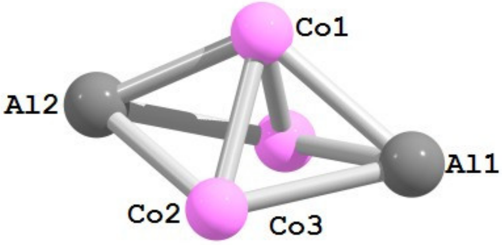

Al_2Co_3 (VI)

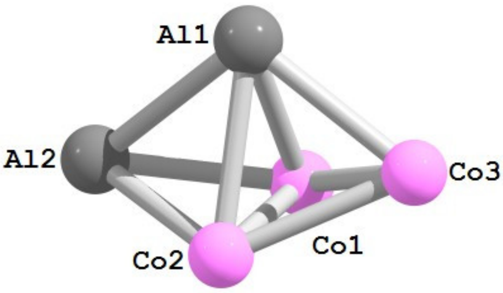

Al_2Co_3 (VII)

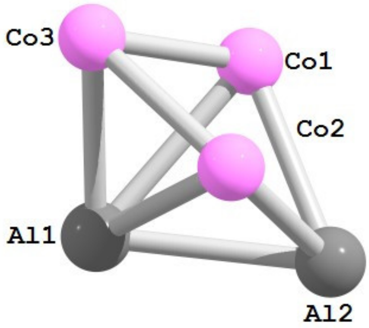

Al_2Co_3 (VIII)

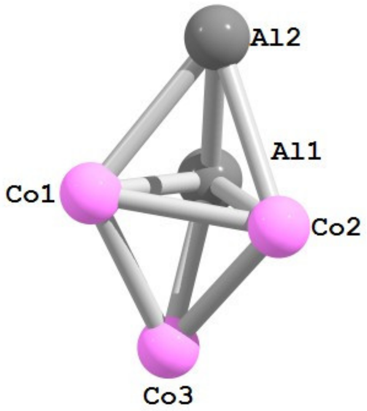

Al_2Co_3 (IX)

Figure S9. Molecular structures of Al_2Co_3 clusters**.**

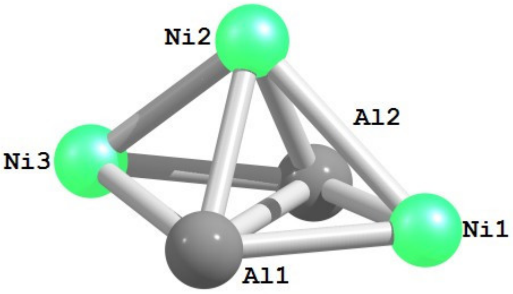

Al_2Ni_3 (I)

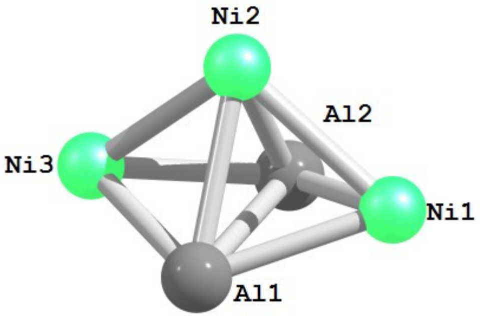

Al_2Ni_3 (II)

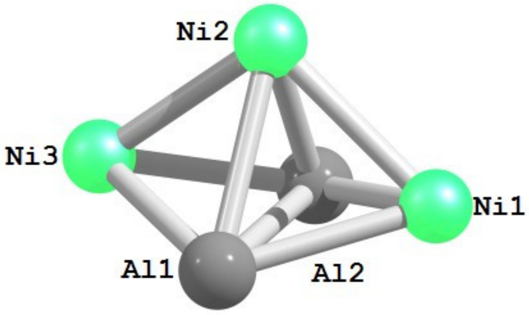

Al_2Ni_3 (III)

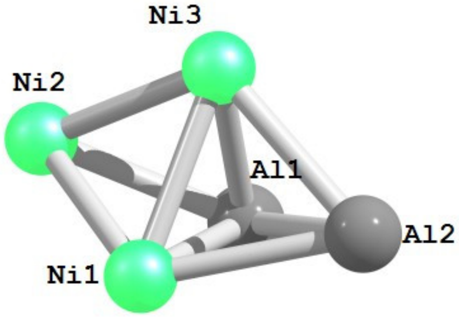

Al_2Ni_3 (IV)

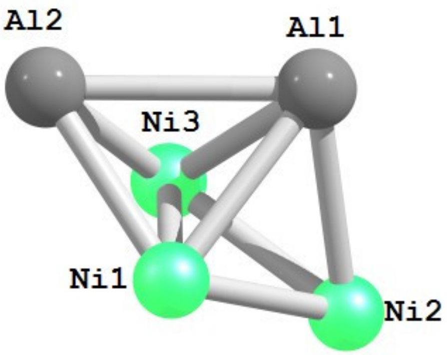

Al_2Ni_3 (V)

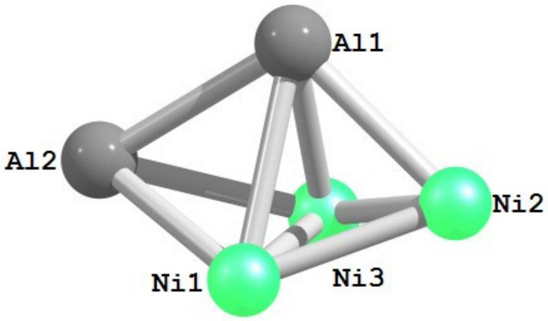

Al_2Ni_3 (VI)

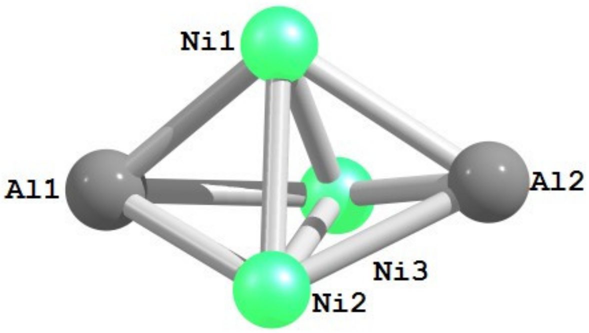

Al_2Ni_3 (VII)

Figure S10. Molecular structures of Al_2Ni_3 clusters.

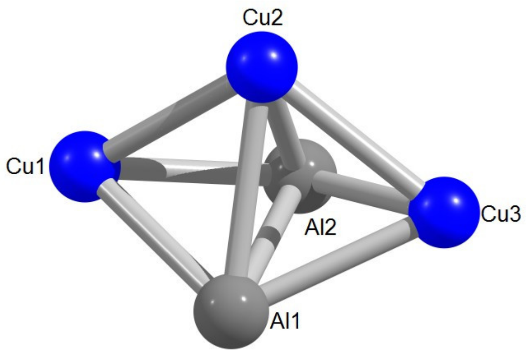

Al_2Cu_3 (I)

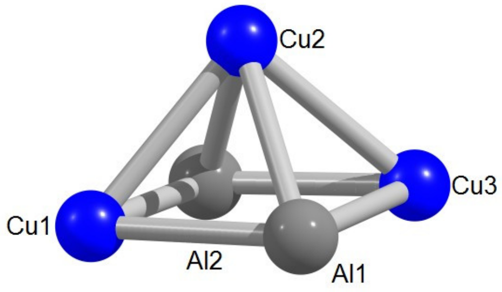

Al_2Cu_3 (I)

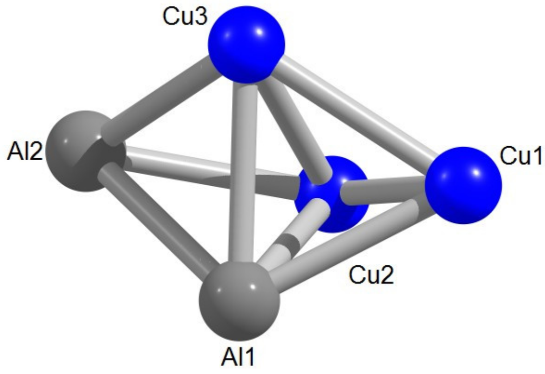

Al_2Cu_3 (III)

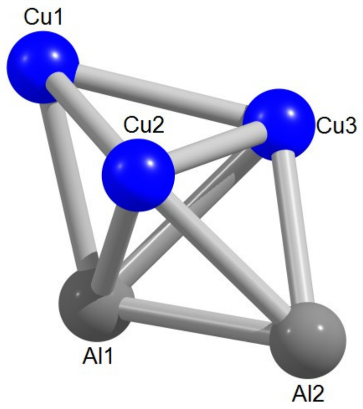

Al_2Cu_3 (IV)

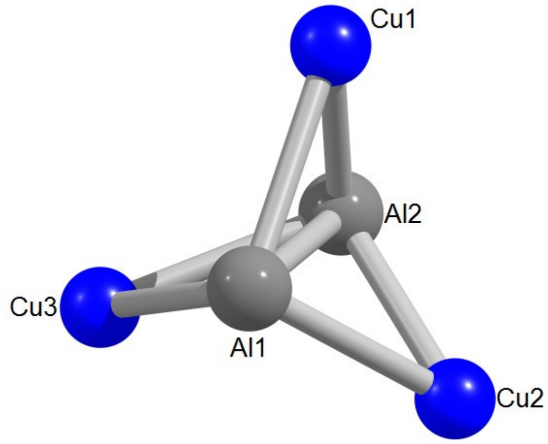

Al_2Cu_3 (V)

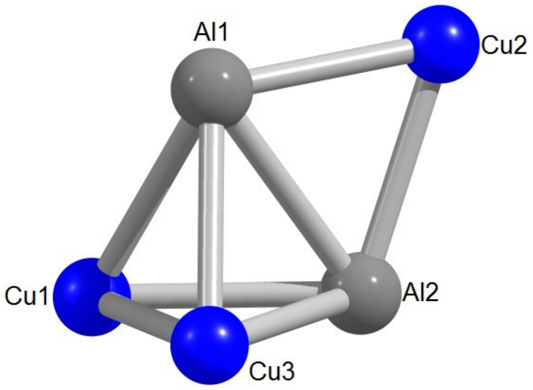

Al_2Cu_3 (VI)

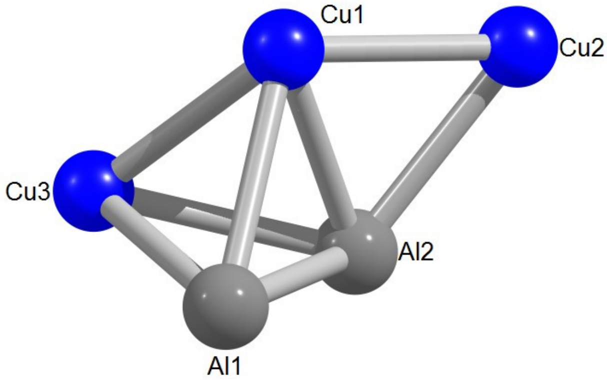

Al_2Cu_3 (VII)

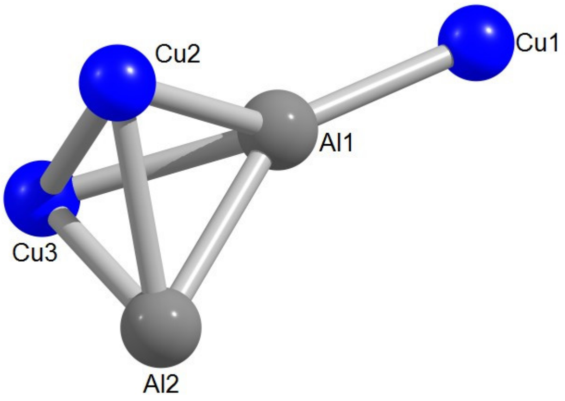

Al_2Cu_3 (VIII)

Figure S11. Molecular structures of Al_2Cu_3 clusters.

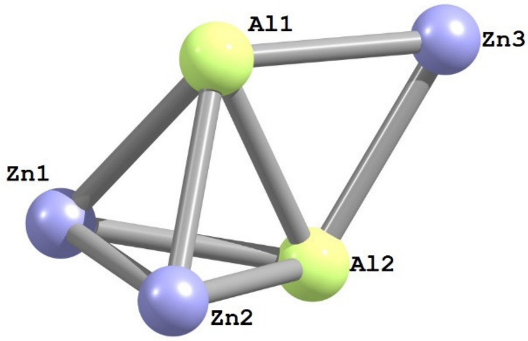

Al_2Zn_3 (I)

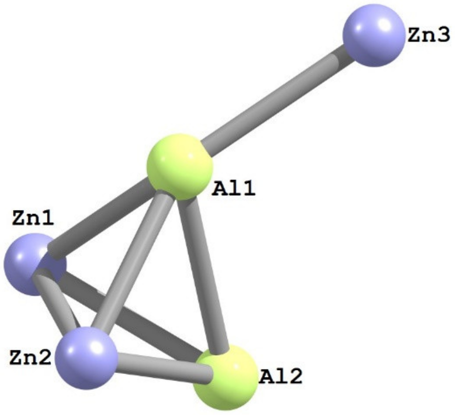

Al_2Zn_3 (II)

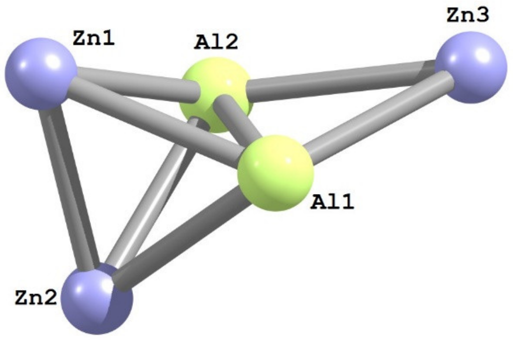

Al_2Zn_3 (III)

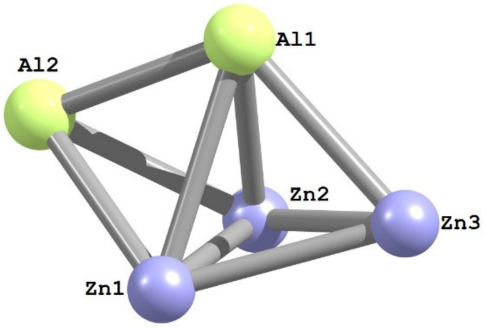

Al_2Zn_3 (IV)

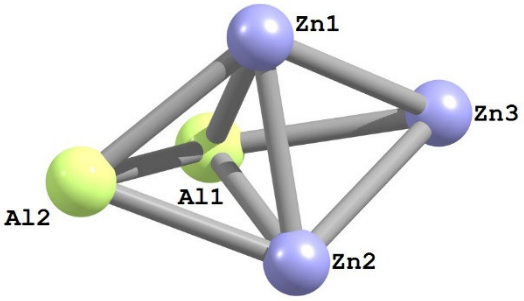

Al_2Zn_3 (V)

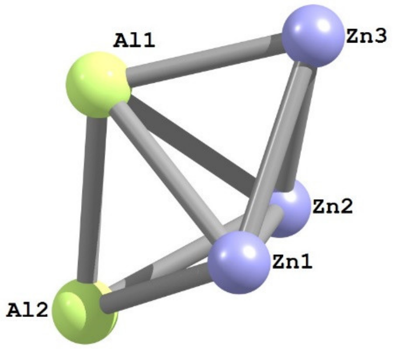

Al_2Zn_3 (VI)

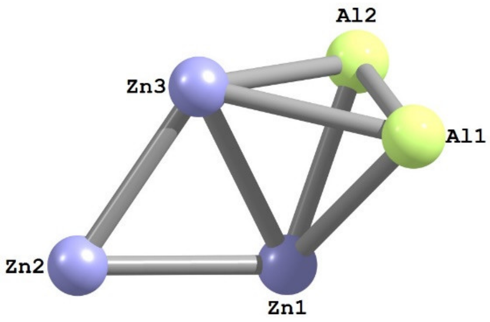

Al_2Zn_3 (VII)

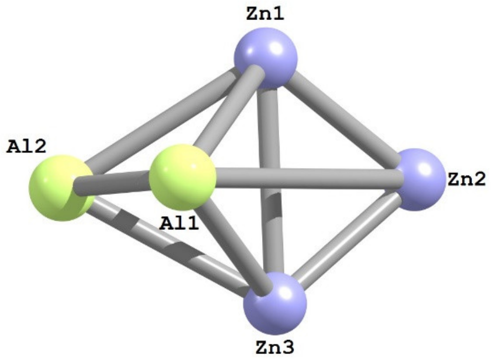

Al_2Zn_3 (VIII)

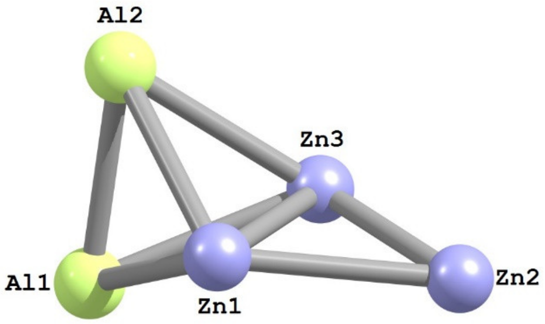

Al_2Zn_3 (IX)

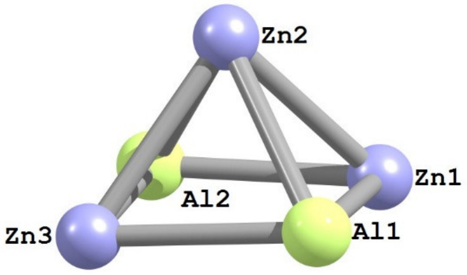

Al_2Zn_3 (X)

Figure 12. (a). Molecular structures of Al_2Zn_3 (I) – Al_2Zn_3 (X) clusters.

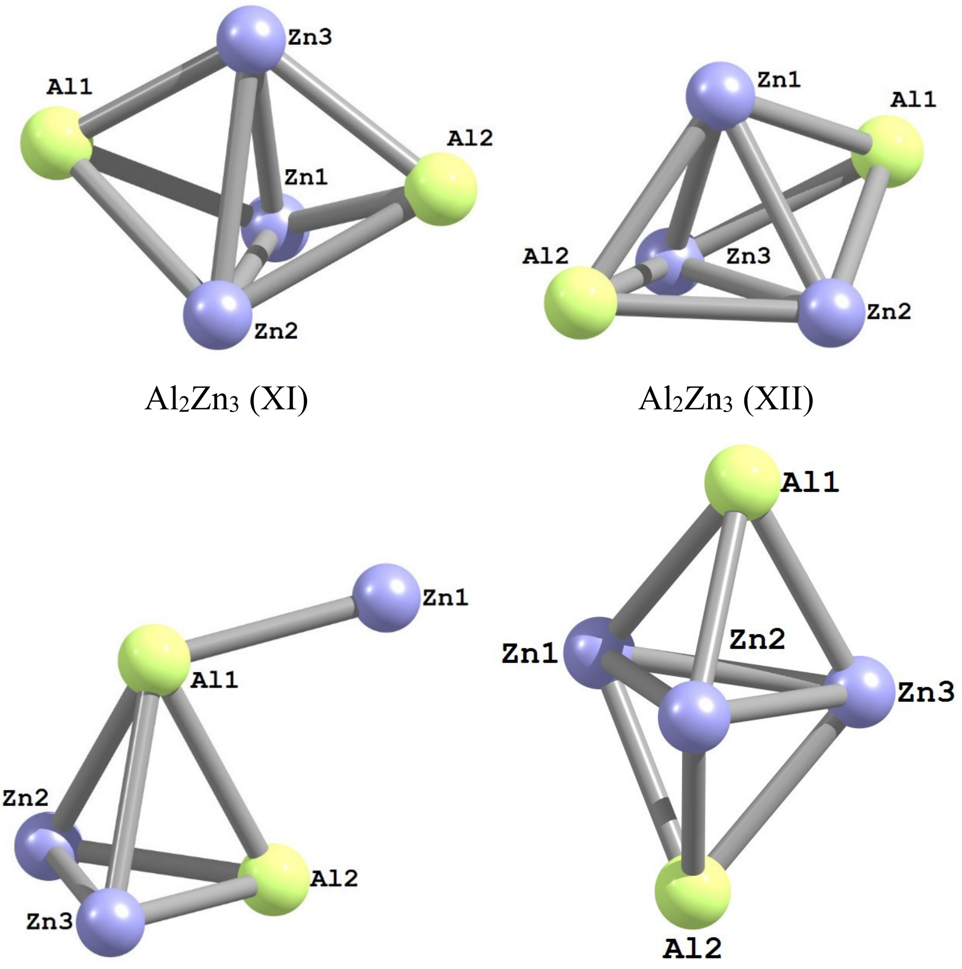

Al_2Zn_3 (XI) Al_2Zn_3 (XII) Al_2Zn_3 (XIII) Al_2Zn_3 (XIV)

Figure 12. (b) Molecular structures of Al_2Zn_3 (XI) – Al_2Zn_3 (XIV) clusters.

Molecular Structures of Hexanuclear Al_3M_3 and Al_2M_4 metal clusters (M= Fe)

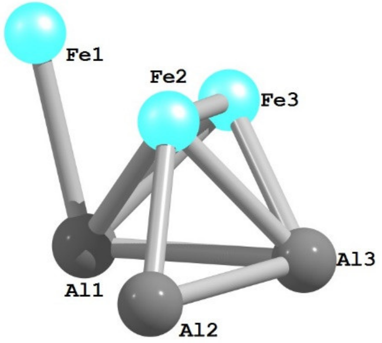

Al_3Fe_3(I)

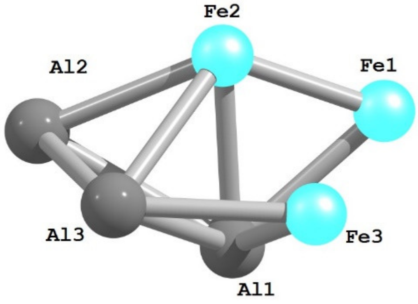

Al_3Fe_3(II)

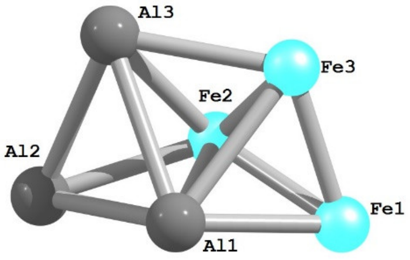

Al_3Fe_3(III)

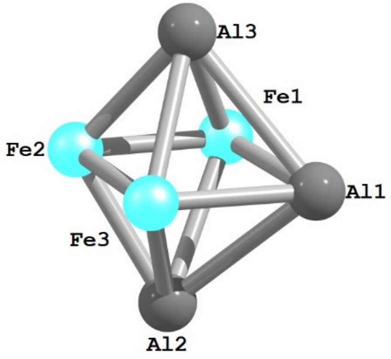

Al_3Fe_3(IV)

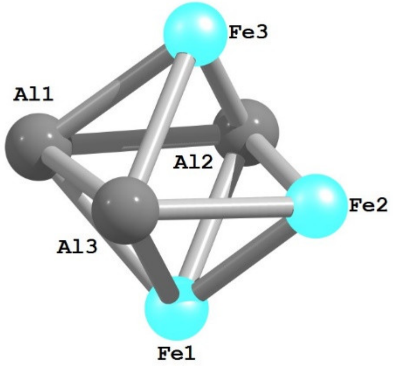

Al_3Fe_3(V)

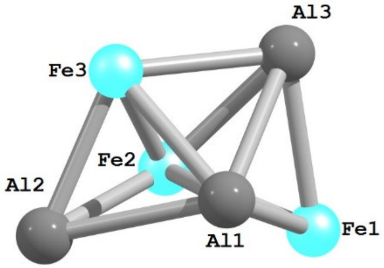

Al_3Fe_3(VI)

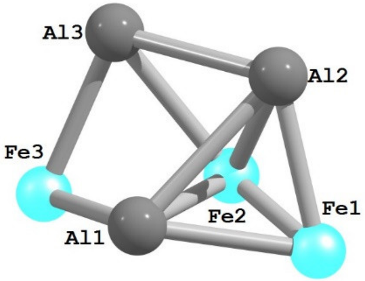

Al_3Fe_3(VII)

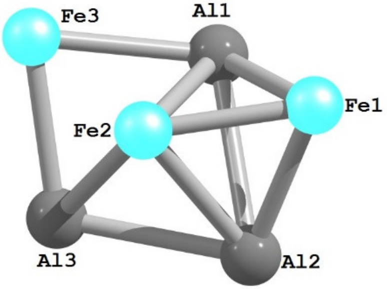

Al_3Fe_3(VIII)

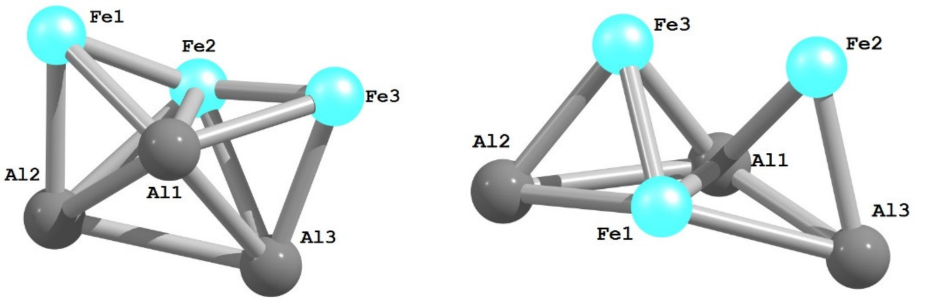

Al_3Fe_3(IX) Al_3Fe_3(X)

Figure S13. (a). Molecular structures of Al_3Fe_3(I) – Al_3Fe_3(X) clusters.

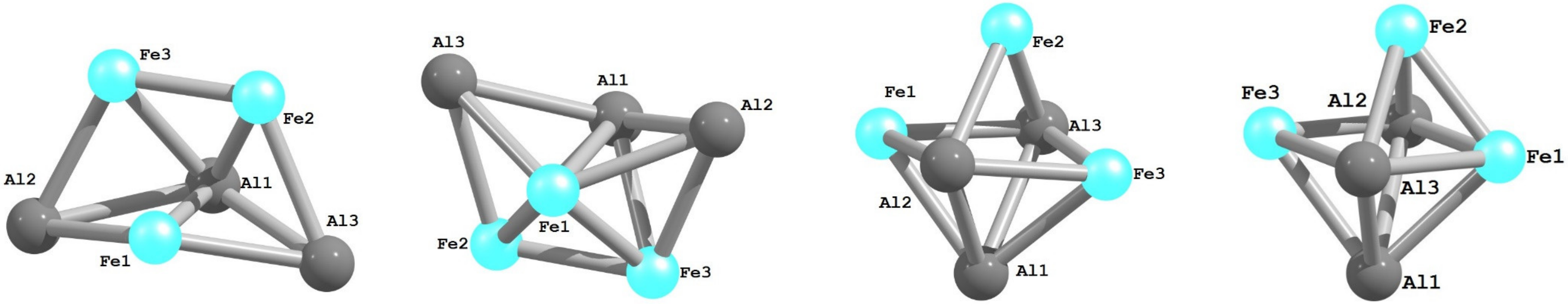

Al_3Fe_3(XI) Al_3Fe_3(XII) Al_3Fe_3(XIII) Al_3Fe_3(XIV)

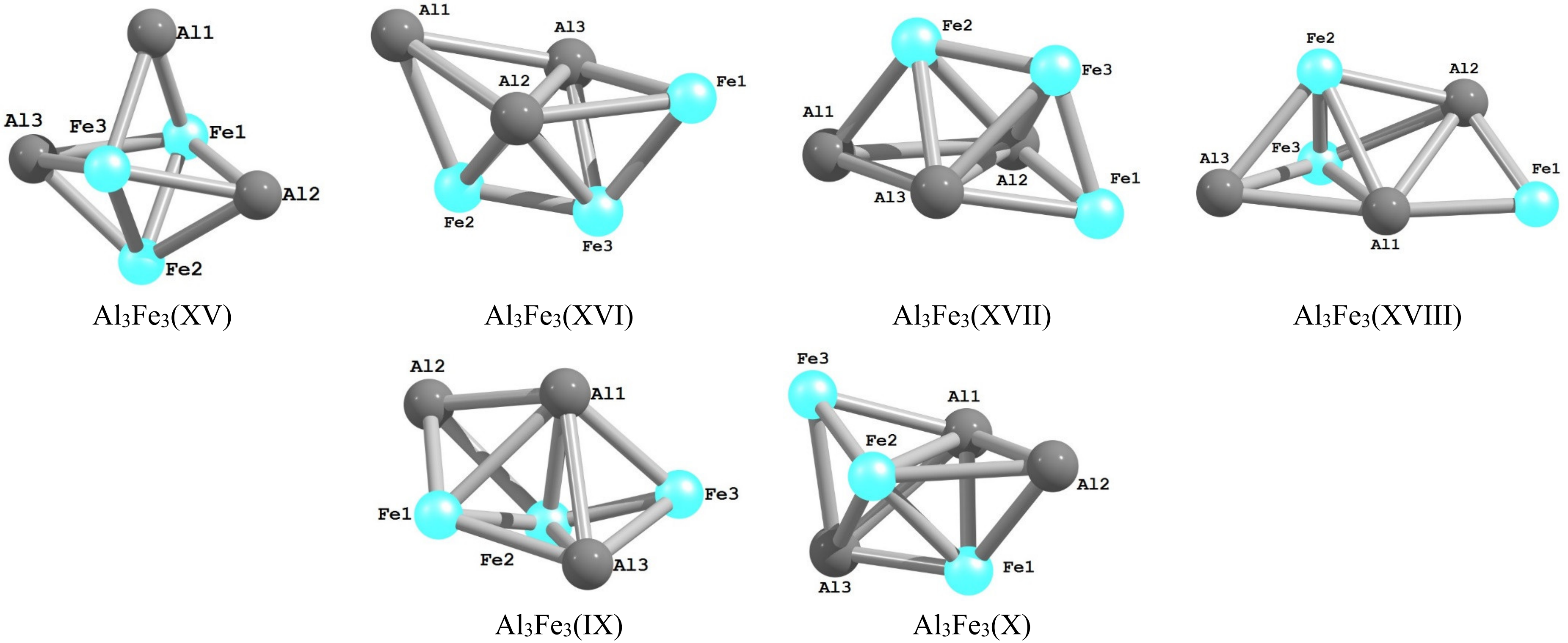

Figure 13. (b). Molecular structures of Al_3Fe_3(XI) – Al_3Fe_3(XX) clusters.

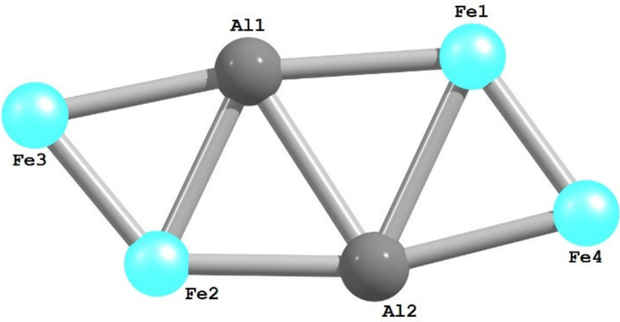

Al_2Fe_4(I)

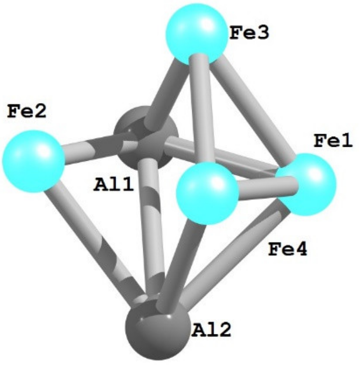

Al_2Fe_4(II)

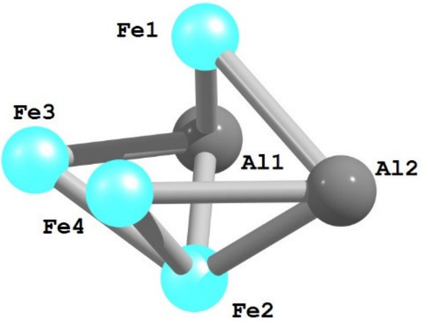

Al_2Fe_4(III)

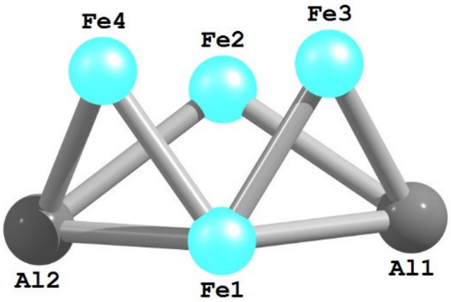

Al_2Fe_4(IV)

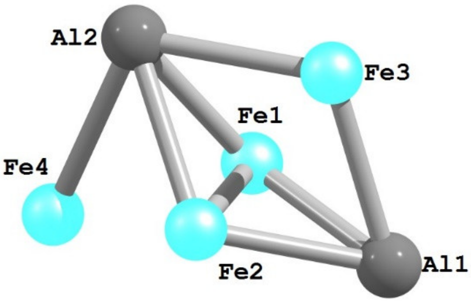

Al_2Fe_4(V)

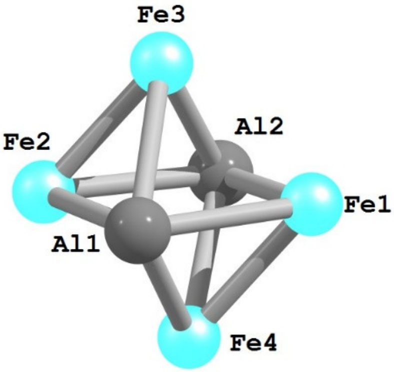

Al_2Fe_4(VI)

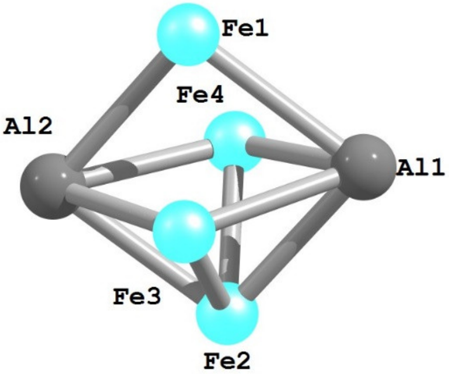

Al_2Fe_4(VII)

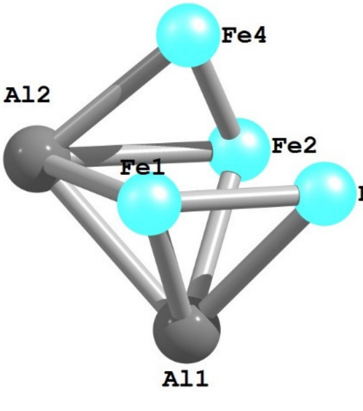

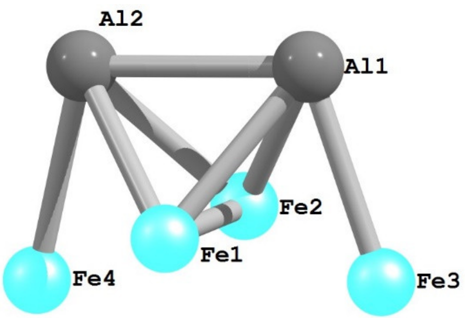

Al_2Fe_4(VIII) Al_2Fe_4(IX)

Figure S14. Molecular structures of Al_2Fe_4 clusters.

© 2020 by the authors. Submitted for possible open access publication under the terms and conditions of the Creative Commons Attribution (CC BY) license (http://creativecommons.org/licenses/by/4.0/).
